# A new regulatory mechanism of protein phosphatase 2A activity via SET in acute myeloid leukemia

**DOI:** 10.1038/s41408-019-0270-0

**Published:** 2020-01-08

**Authors:** Elena Arriazu, Carmen Vicente, Raffaella Pippa, Irene Peris, Elena Martínez-Balsalobre, Patricia García-Ramírez, Nerea Marcotegui, Ana Igea, Diego Alignani, José Rifón, María C. Mateos, María L. Cayuela, Angel R. Nebreda, María D. Odero

**Affiliations:** 1 University of Navarra, Centro de Investigación Médica Aplicada (CIMA), Pamplona, Spain; 2 CIBERONC, Instituto de Salud Carlos III, Madrid, Spain; 3 University of Navarra, Biochemistry and Genetics Department, Pamplona, Spain; 4 Thomas Jefferson University, Sidney Kimmel Cancer Center, Molecular Oncology Department, Philadelphia, USA; 5 University Hospital Virgen de la Arrixaca, and Instituto Murciano de Investigación Biosanitaria (IMIB), Murcia, Spain; 6 Hematology Service, Complejo Hospitalario de Navarra (CHN), Pamplona, Spain; 7 Institute for Research in Biomedicine (IRB Barcelona), Barcelona Institute of Science and Technology, Barcelona, Spain; 8 IdiSNA, Instituto de Investigación Sanitaria de Navarra, Pamplona, Spain; 9 Catalan Institution for Research and Advanced Studies (ICREA), Barcelona, Spain

**Keywords:** Biochemistry, Cell signalling, Acute myeloid leukaemia, Acute myeloid leukaemia

## Abstract

Acute myeloid leukemia (AML) is an aggressive hematologic malignancy. Although novel emerging drugs are available, the overall prognosis remains poor and new therapeutic approaches are required. PP2A phosphatase is a key regulator of cell homeostasis and is recurrently inactivated in AML. The anticancer activity of several PP2A-activating drugs (e.g., FTY720) depends on their interaction with the SET oncoprotein, an endogenous PP2A inhibitor that is overexpressed in 30% of AML cases. Elucidation of SET regulatory mechanisms may therefore provide novel targeted therapies for *SET*-overexpressing AMLs. Here, we show that upregulation of protein kinase p38β is a common event in AML. We provide evidence that p38β potentiates SET-mediated PP2A inactivation by two mechanisms: facilitating SET cytoplasmic translocation through CK2 phosphorylation, and directly binding to and stabilizing the SET protein. We demonstrate the importance of this new regulatory mechanism in primary AML cells from patients and in zebrafish xenograft models. Accordingly, combination of the CK2 inhibitor CX-4945, which retains SET in the nucleus, and FTY720, which disrupts the SET-PP2A binding in the cytoplasm, significantly reduces the viability and migration of AML cells. In conclusion, we show that the p38β/CK2/SET axis represents a new potential therapeutic pathway in AML patients with SET-dependent PP2A inactivation.

## Introduction

Acute myeloid leukemia (AML) is a highly heterogeneous fatal disease that results from the enhanced proliferation and impaired differentiation of hematopoietic stem and progenitor cells^1^. For decades, chemotherapy consisting of cytarabine and anthracyclines has been the standard in AML care. Emerging drugs show promising results^2,3^; however, the outcome for AML remains poor and most patients ultimately relapse and die from disease progression despite initial sensitivity to chemotherapy. Patients older than 60 years old, who represent the main group, are refractory to cytotoxic intensive chemotherapy because of biological disease-related factors, such as increased frequency of adverse-risk cytogenetic and molecular features, and secondary AML^3^. Moreover, they present comorbidities that reduce their tolerance of intensive therapies, leaving few treatment options in most cases^1^. Even in younger patients the outcome is dismal. In patients ≤60 years old complete remission is achieved in around 70%, but a subset of patients relapse, depending on the prognostic factors, and only 5–10% survive after relapse^1,4^. Current efforts directed towards the genetic characterization of AML have led to the development of new targeted therapies, including FLT3, BCL2 and IDH1/2 inhibitors^5–10^. However, monotherapy with these drugs does not result in durable responses. Thus, further research is necessary to develop new personalized therapeutic strategies for the treatment of this aggressive disease.

Reversible phosphorylation allows the cell to maintain a proper homeostasis regulation; therefore, the balance between kinases and phosphatases is essential to control correct proliferation, apoptosis, and differentiation. Many studies have analyzed the abnormal behavior of protein kinases in AML, but the role of phosphatases remains underexplored^11,12^. Protein phosphatase 2A (PP2A) is a tumor suppressor that regulates several essential cell functions and counteracts most of the kinase-driven intracellular signaling pathways^13^. Previous results from our group and others showed that PP2A inactivation is a recurrent event in AML, and that its pharmacological activation by PP2A-activating drugs (OP499, FTY720, and its analogues) effectively antagonizes leukemogenesis^14,15^. Furthermore, preclinical studies show that these drugs have synergistic effects with conventional chemotherapy and tyrosine kinase inhibitors, opening new possibilities for personalized medicine in AML^16,17^. Interestingly, the anticancer activity of several PP2A-activating drugs depends on their ability to interact with the endogenous PP2A inhibitor SET, an oncoprotein overexpressed in ~30% of AML patients and associated with poor outcome^18,19^. Therefore, targeting SET allows PP2A to be reactivated indirectly, avoiding toxicity problems related to the direct activation of this complex holoenzyme. SET is a multitask oncogenic protein involved in many cellular processes^20–23^. However, despite the prognostic impact of SET overexpression in both hematologic and solid tumors, the mechanisms by which SET is regulated remain poorly understood. We have previously reported a novel multi-protein complex that activates *SET* transcription in AML^24^. Here, we explore the post-transcriptional regulation of SET, which may help us to develop novel targeted therapies in AML patients with PP2A inactivation and high expression of *SET*. Using genetic and pharmacological approaches, we found that p38β, one of the p38 family members whose function is not well known, has a dual role in the regulation of PP2A activity in AML. p38β regulates SET phosphorylation and intracellular localization through the activity of casein kinase 2 (CK2). Furthermore, p38β stabilizes the SET protein, facilitating its PP2A inhibitory role. Importantly, we validated this mechanism in vivo by demonstrating that the combination of the CK2 inhibitor CX-4945, and the PP2A-activating drug FTY720 significantly reduces the viability and migration of AML cells. This novel mechanism may constitute the basis for targeted therapy in AML patients with SET overexpression.

## Materials and methods

### Patient samples

The study comprised peripheral blood mononuclear cells (PB-MC) samples of 27 patients with AML at diagnosis who stated an informed consent (Supplementary Table S1). All patients were treated with standard induction chemotherapy. High-dose cytarabine, and autologous or allogeneic stem cell transplantation, when possible, were used as consolidation therapy. PB-MC samples of healthy donors were used as controls. This study is part of a project approved by the *Comité Ético de Investigación Clínica, Gobierno de Navarra* (2018/32). The experiments conformed to the principles set out in the WMA Declaration of Helsinki. AML patient sample cells (CD34^+^) were cultivated in the semisolid medium MethoCult (StemCell Technologies, Grenoble, France) supplemented with penicillin G (100 U/ml) and streptomycin (0.1 mg/ml). In the medium, different concentrations of FTY720, CX-4945 and combination were added. After 12–14 days growing at 37 °C in a 5% CO_2_ atmosphere, the present colonies were counted at an inverted light microscope (Leica Biosystems, Barcelona, Spain) using a grid (2700, StemCell).

### In vitro kinase assay

Bacterially-expressed p38α or p38β (0.2 µg) were pre-incubated with purified MKK6 (40 ng) and then incubated with purified GST, GST-ATF2 or GST-SET (1 µg) in kinase buffer (50 mM Tris-HCl pH 7.5, 10 mM MgCl_2_, 2 mM DTT, 0.1 mM Na_3_VO_4_, 1 mM PMSF and 10 µg/ml aprotinin and leupeptin) containing 100 μM cold ATP and 2μCi of [γ-^32^P]ATP (3 000 Ci/mmol) for 40 min at 30 °C. Reactions were stopped by adding sample loading buffer and boiling 5 min. Proteins were resolved by SDS-PAGE, stained with Coomassie, and analyzed by autoradiography.

### Plasmids, siRNA, and transfection

siRNAs were from Ambion (Madrid, Spain): scramble siRNA *(#AM4635)*, MAPK11/p38β (#1:s11155 and #2:11156), MAPK14/p38α (#1:s3586 and #2:s3585), and CK2 (s3638). SET siRNAs were *siSET#1 (#23-2506-2/4, Eurofins*, Ebersberg, Germany*) and siSET#2 (#5883466, Invitrogen)*. Due to the high efficiency obtained with siRNAs #1 from p38α and p38β we used them for all experiments. For silencing experiments, cells were transfected using GenePulser Xcell^TM^ (Bio-Rad, Madrid, Spain) with 300 V and 1000 µF. The shRNAp38β cloned in the pINDUCER 11 (44.363 from Adgene, Teddington, UK) was shRNA1: CACGTTCAATTCCTGGTTT and shRNA2: GCGCCAGAAGGTGGCGGTGAAG.

### General methodology

Details on general methodology as western blot, protein immunoprecipitation, apoptosis, and MTS assay have been previously described^14,18,19,24,25^. Reagents and antibodies used are displayed in Supplementary Tables S2 and S3, respectively. Nuclear and cytoplasmic proteins were extracted using the NE-PER nuclear and cytoplasmic extraction kit (Thermo-scientific, UK) according to manufactured instructions.

### Cell culture and treatments

HL60, MOLM-13, and HEK293T cells were maintained in RPMI-1640 (Invitrogen, UK) supplemented with 10% FBS, penicillin G (100U/ml) and streptomycin (0.1 mg/ml). Cell lines were grown at 37 °C in a 5% CO_2_ atmosphere. Prior treatments, cells were plated at 100,000 cells/ml.

### Immunofluorescence

100,000 cells were seeded on cover slips coated with poly-L-lysine (Sigma, Madrid, Spain), fixed with 4% paraformaldehyde (Thermo-scientific) and permeabilized with 0.1% Triton-X-100. After blocking with 5% FBS, incubation with primary and secondary antibodies were performed (Supplementary Table S3). Images were acquired using a Confocal Scanning Laser Microscopy Zeiss LSM 800 with ×63 immersion oil objective. Image quantification was performed using Fiji software^26^. For colocalization, the red, green and red-green colocalization volumes (umÂ³) were quantified and referred to total cell volume. For nuclear and cytoplasmic quantification, green volume (umÂ³) was measured in the cytoplasm and the nucleus and referred to total cell volume.

### Phos-tag, immunoblot and λ-phosphatase treatment

10% acrylamide gels were prepared in the presence of 40 µM of Phos-tag (Fujifilm Wako, Neuss, Germany) and 20 µM of MnCl_2_. Proteins were transferred to a PVDF membrane (Immobilon-P membranes, Millipore, Madrid, Spain) using the Tank blotting system (Bio-Rad, Madrid, Spain). As a control, an aliquot of the cell lysate (15 µg) was incubated with 100 units of λ-phosphatase (Biolabs, Spain) for 1 h at 30 °C in a shaking thermoblock.

### Migration assay

Migration assay was performed in a 24-transwell permeable plate with 8.0 μM pores (Corning Costar, Madrid, Spain). The lower compartment contained RPMI supplemented with 10% FBS. 500,000 treated cells were seeded in the upper insert in medium without serum and allowed to migrate for 3 h. The volume of the bottom well was collected and mixed with perfect-count microspheres (cytognos, Salamanca, Spain). The amount of viable migrated cells was determined by flow cytometry, counting 5000 microsphere-events and expressed as a percentage of the control.

### Zebrafish husbandry and embryo collection

Wild-type zebrafish (*Danio rerio*, AB strain), from the Zebrafish International Resource Centre, were maintained in re-circulating tanks according to the standard procedures. Adult fishes were maintained at 26 °C, with a light/dark cycle of 14/10 h, and were fed twice daily, once with dry flake food (Prodac, Italy) and another with live *Artemia salina* (MC 450, Ive Aquaculture, USA). Zebrafish embryos were maintained in egg water at 28.5 °C, fed for 5 days with Novo Tom and with live *Artemia salina* at 11 days of life. All experiments were performed in compliance with the Guidelines of the European Union Council for animal experimentation (86/609/EU).

### Xenograft of human leukemia cells into zebrafish embryos

Wild-type zebrafish embryos at 48hpf were anesthetized with 0.04% Tricaine (Sigma–Aldrich). Treated leukemia cells were stained with red fluorescent CM-DiI (Invitrogen) prior the injection. 50–75 labeled cells were injected into the yolk sac of dechorionated zebrafish embryos using a manual injector (Narishige). Fish with fluorescently labeled cells appearing outside the implantation area at 2hpi were excluded from analysis. All other fishes were incubated at 35 °C for 72 h and analyzed with the SteReo Lumar V12 stereomicroscope with an AxioCam MR5 camera (Carl Zeiss, Germany). Positive embryo colonization was considered when more than five human leukemia cells were present outside the yolk sac at 72hpx. Zebrafish colonization index was calculated as the proportion of embryos colonized in the treatment condition divided by the proportion of invaded embryos in the control condition. Tumor growth and proliferation were evaluated at 2 (reference) and 72hpx in a M205-FA fluorescence microscopy with a DFC365FX camera (Fujifilm Leica). Proliferation index (Fluorescence intensity medium value*fluorescence pixel number) and area were measured with a Leica Application Suite-X software.

### Statistical analysis

Data represented are the mean of three independent experiments ±S.D. Statistical comparisons were carried out using the nonparametric method Kruskal–Wallis test for more than two independent samples, followed by Mann–Whitney U test to compared two groups when the distribution was not normal (Shapiro-Wilk test *p* < 0,05). Two-way ANOVA (Tukey’s multiple comparisons test) when the distribution was normal (zebrafish proliferation experiments). Chi-square statistical analysis was done for the invasive potential calculation in zebrafish experiments. Significance was considered when *p* < 0.05. For AML patient samples tested, 20% decrease in viability was chosen as the threshold as a response to the treatments.

## Results

### p38β overexpression regulates PP2A activity in AML through SET

**Fig. 1:**
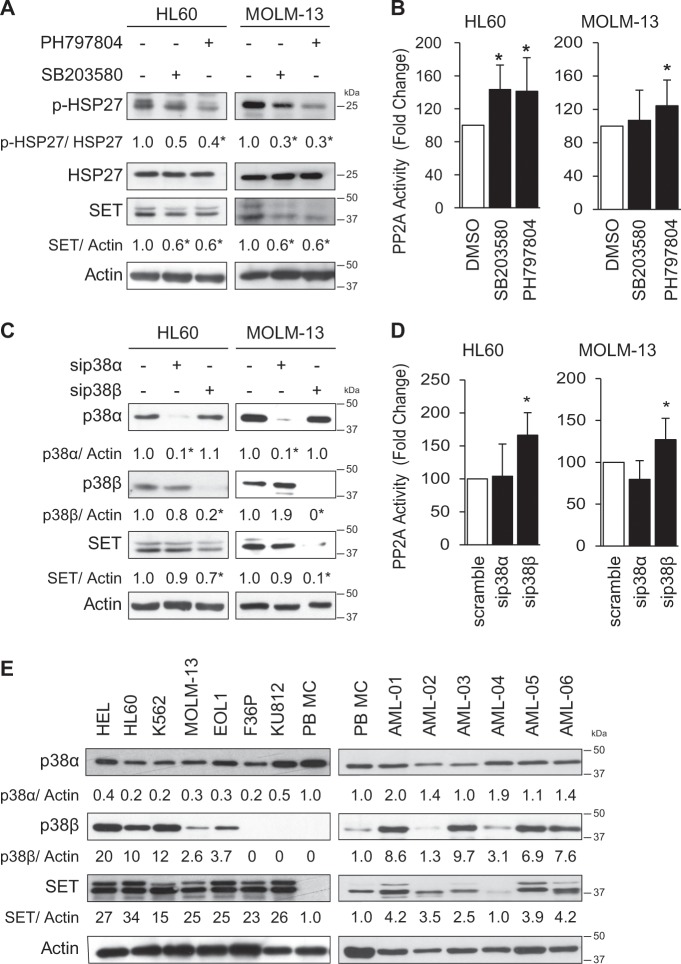
p38β is overexpressed in AML and its inhibition decreases SET protein levels, increasing PP2A activity. **a** HL60 and MOLM-13 cell lines were treated with the p38 inhibitors SB203580 (2.5 µM) and PH797804 (250 nM) for 24 h. Protein expression for p-HSP27/HSP27 (p38 substrate) and SET was analyzed by western blot. **b** Measurement of PP2A activity after p38 inhibition by immunoprecipitation and phosphatase assay. **c** Silencing of p38α and p38β with specific siRNA (50 nM for 48 h), and analysis of total protein by western blot. **d** Measurement of PP2A activity by immunoprecipitation and phosphatase assay. **e** Western blot analysis of total protein in AML cell lines and AML patient samples, compared to peripheral blood mononuclear cells (PB-MC). The results are corrected by the specific loading control and are expressed as fold-change of the control, which are assigned a value of 1 and are mean values. Experiments were performed in triplicate four times. *p < 0.05.

To investigate the regulation of the SET oncoprotein in AML, we performed a functional drug screen using inhibitors of the main signaling pathways such as PI3K, p38, JNK, and ERK in HL60 cells. Notably, only p38 inhibition using either SB203580 or PH797804 decreased SET protein content without altering its mRNA levels (Supplementary Fig. S1a, b), suggesting that SET was regulated at post-transcriptional level. In fact, reduced phosphorylation of HSP27, a downstream target of p38, paralleled the decrease in SET protein levels (Fig. 1a). Since SET is an important inhibitor of PP2A in AML, we assessed the PP2A activity in the treated cells. As expected, both p38 inhibitors increased PP2A activity (Fig. 1b and Supplementary Fig. S1a), suggesting that p38 inhibition affects PP2A activity in AML through SET. These results were confirmed in MOLM-13, another AML cell line (Fig. 1 and Supplementary Fig. S1c).

The p38 family has four members: p38α, p38β, p38γ, and p38δ. As p38α and p38β are the main targets of SB203580 and PH797804 at the tested concentrations^27,28^, we focused on these two kinases. Knockdown of p38α or p38β by specific siRNAs showed that downregulation of p38β, but not p38α, significantly decreased SET protein levels and increased PP2A activity (Fig. 1c, d).

To explore the clinical relevance of this finding, we assessed p38α and p38β expression in AML. The p38β protein was highly expressed in 5 out of 7 AML cell lines (71%), and in 23 out of 27 AML patient samples (85%); whereas the p38α protein was almost equally expressed in PB and AML specimens (Fig. 1e, Supplementary Fig. S2). Correlation analysis indicated a positive co-expression between SET and p38β protein levels, which was statistically significant (R^2^ = 0.376 p-value 0.0014). However, no correlation was found between p38α and SET (R^2^ = 0.004 p-value 0.7694). Quantitative analysis confirmed that p38α was expressed at similar levels in PB and AML cell lines (20–30 ng/100 µg total protein). However, p38β was expressed at lower levels than p38α in HL60 and MOLM-13 cells (2–3 ng/100 µg total protein), but it was undetectable in PBMC (Supplementary Fig. S3). Taken together, these results suggest that p38β is overexpressed in AML and can regulate PP2A activity via SET.

### p38β binds to and stabilizes SET in AML cells

**Fig. 2:**
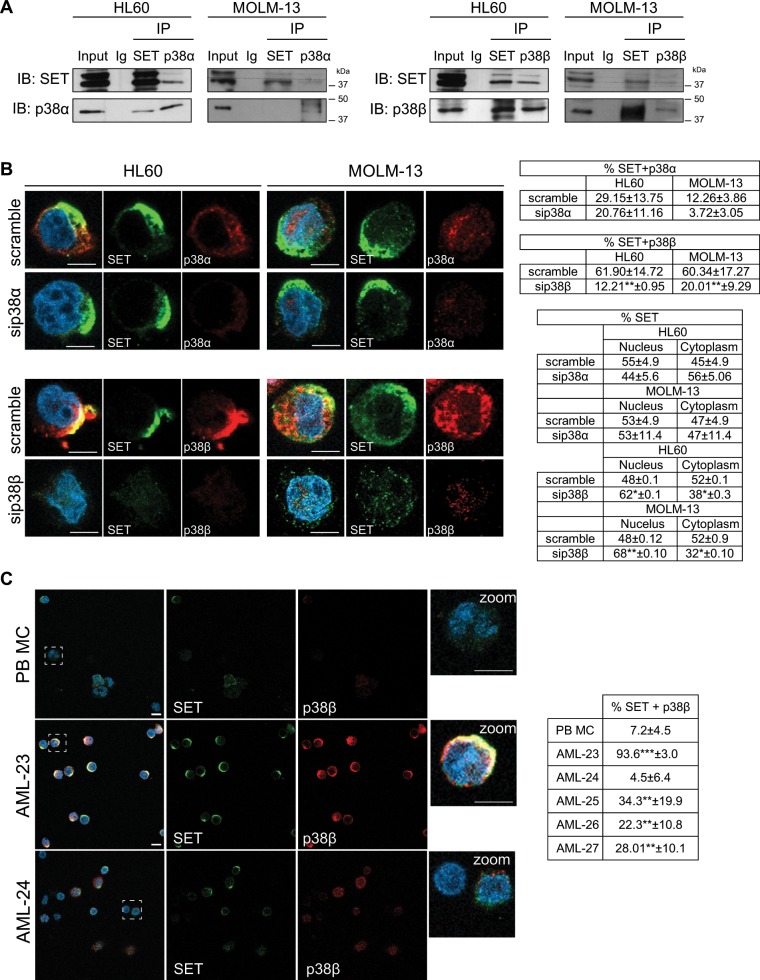
p38β co-localizes with SET in AML cells. **a** Immunoprecipitation of SET, p38α and p38β with specific antibodies in HL60 and MOLM-13 cells. Normal goat Ig was used as negative control **b** Knockdown of p38α and p38β with siRNA (50 nM for 48 h), using scramble siRNA as control, in HL60 and MOLM-13 cells. Immunofluorescence analysis of either p38α or p38β (red) and SET (green). Nuclei were stained with DAPI. Immunofluorescences were visualized by confocal microscopy. Quantification table of colocalization fluorescence and green fluorescence (SET) in nucleus and cytoplasm. Quantification analysis showed ~60% of SET-p38β colocalization in both cell lines, and only 12 and 29% of SET-p38α colocalization in MOLM-13 and HL60, respectively. **c** Immunofluorescence analysis of p38β (red) and SET (green) in peripheral blood mononuclear cells (PB-MC) and the primary AML patient samples AML-23 and AML-24. Nuclei were stained with DAPI. Quantification table of colocalization fluorescence. Immunofluorescences were visualized by confocal microscopy. The results are expressed as mean values ± SEM. Experiments were performed in triplicate four times. *p < 0.05, **p < 0.01. Scale bar represents 5 µm.

We next focus on dissecting the mechanisms through which p38β regulates the SET protein. Co-immunoprecipitation experiments indicated that SET bound to p38β in both HL60 and MOLM-13 cells, and to a lesser extent, to p38α in HL60 cells (Fig. 2a). Immunofluorescence analysis confirmed high expression and cytoplasmic colocalization between p38β and SET, which disappeared after silencing p38β, whereas p38α silencing had no effect (Fig. 2b).

**Fig. 3:**
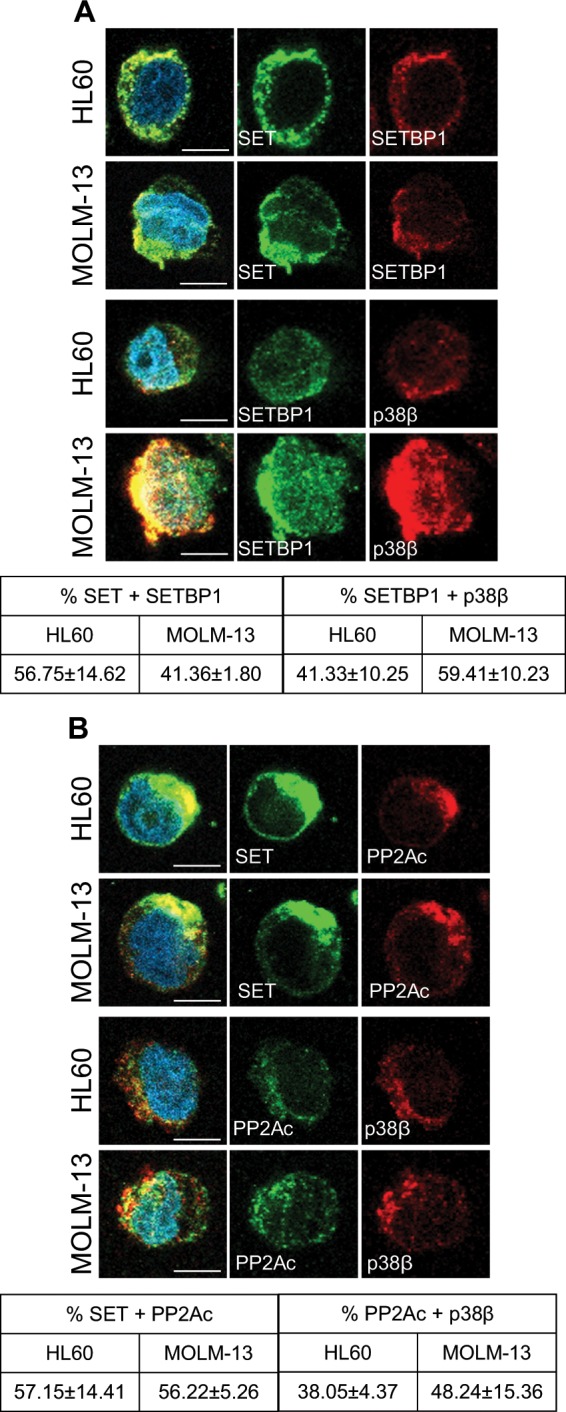
p38β acts as a SET stabilizing protein. **a** Immunofluorescence analysis of SET (green) and SETBP1 (red), and SETBP1 (green) and p38β (red), in HL60 and MOLM-13 cells. Nuclei were stained with DAPI. Quantification of colocalization fluorescence. **(b)** Immunofluorescence analysis of SET (green) and PP2Ac (red) and PP2Ac (green) and p38β (red), in HL60 and MOLM-13 cells. Nuclei were stained with DAPI. Quantification of colocalization fluorescence. Immunofluorescences were visualized by confocal microscopy. The results are expressed as mean values ± SEM. Experiments were performed in triplicate four times. *p < 0.05, **p < 0.01. Scale bar represents 5 µm.

We hypothesized that p38β might phosphorylate SET. Surprisingly, in vitro kinase assays showed no direct SET phosphorylation either by p38β or p38α (Supplementary Fig. S4a). Importantly, co-immunoprecipitation in HL60 cells treated with p38 inhibitors showed that SET-p38 interaction did not require kinase activation (Supplementary Fig. S4b). For these reasons, we postulated that p38β could regulate SET stability in a kinase-independent manner. Treatment of cells with cycloheximide demonstrated that SET is stable up to 48 h (Supplementary Fig. S5a). Treatment of p38β-silenced cells with cycloheximide resulted in a significant decrease in SET (Supplementary Fig. S5b), suggesting that p38β-SET interaction is critical for SET stability. Besides, immunofluorescence analysis in samples from AML patients that overexpress SET and p38β, such as AML-23 or AML-25, demonstrated that both proteins tend to associate and colocalized in the cytoplasm. In contrast, samples from patients with no SET or p38β overexpression, such as AML-24, showed minimal colocalization (Fig. 2c, Supplementary Fig. S6a, b). These results support the biological importance of SET-p38β binding in AML, and suggest that p38β contributes to cytoplasmic SET stability. Data from our group previously reported that SET protein stability is enhanced through its binding to SETBP1^25^. Here, we show that SETBP1 and p38β colocalized along with SET in the cytoplasm (Fig. 3a, Supplementary Fig. S7). Furthermore, we found SET-PP2Ac and PP2Ac-p38β colocalization and interaction in AML cells (Fig. 3b). Taken together, these results suggest that p38β acts as a SET stabilizing protein, together with SETBP1, allowing SET to inhibit PP2A in the cytoplasm.

### p38β regulates CK2-mediated phosphorylation of SET and facilitates its translocation to the cytoplasm

**Fig. 4:**
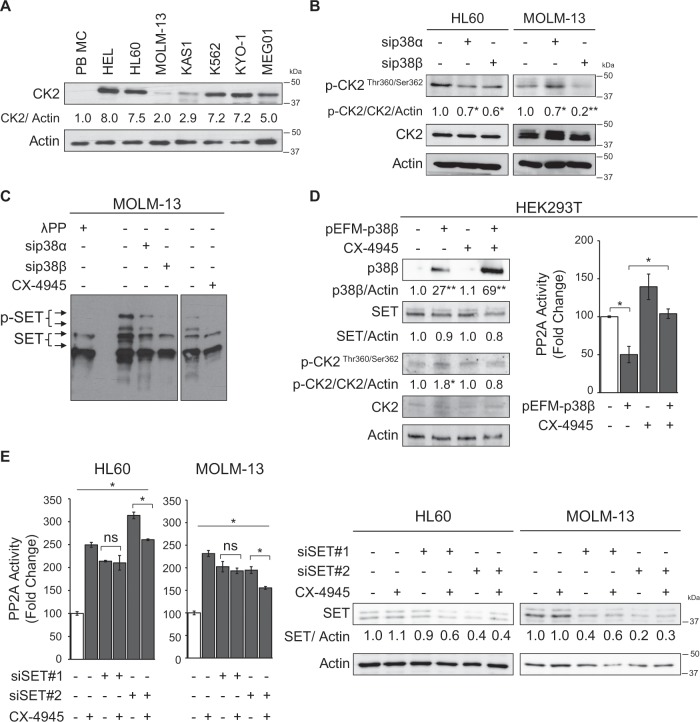
p38β regulates CK2-mediated phosphorylation of SET. **a** Western blot analysis of the CK2 protein in AML cells lines compared to peripheral blood mononuclear cells (PB-MC). **b** Silencing of p38α and p38β with specific siRNA (50 nM for 48 h) and analysis of phospho- and total CK2 by western blot in HL60 and MOLM-13 cells. **c** MOLM-13 cells treated with either siRNA for silencing p38α and p38β (50 nM for 48 h) or with the CK2 inhibitor CX-4945 (5 µM, 24 h) and analyzed for phosphorylated forms of SET in SDS-PAGE with Phos-Tag^TM^. A sample treated with λ phosphatase (100 units, 1 h) was used as control. **d** Overexpression of p38β in HEK293T cells *with* 1 µg of pEFM link p38β plasmid or the empty plasmid with lipofectamine 2000 and treated with CX-4945 (3,75 µM, 24 h). Analysis of p38β, SET and phospho- and total CK2 by western blot and PP2A activity. **e** Silencing of SET with specific siRNA (50 nM for 72 h) and analysis of SET by western blot and PP2A activity in HL60 and MOLM-13 cells. The results are corrected by the specific loading control and are expressed as fold-change of the control, which are assigned a value of 1 and are mean values. Experiments were performed in triplicate four times. *p < 0.05 **p < 0.01.

SET is mainly localized in the nucleus^29^, but AML cells overexpressing SET showed strong cytoplasmic half-moon-shape localization (Fig. 2b, c). It has been reported in Alzheimer’s disease models that CK2 phosphorylates Ser9 on SET, leading to its cytoplasmic translocation and inhibition of PP2A, resulting in tau phosphorylation^30,31^. CK2 is overexpressed in most hematological tumors, including AML^32^, and it is a target of p38 signaling^33^. This data prompted us to postulate the potential role of p38β in regulating CK2 and, consequently, the phosphorylation of SET in AML. Western blot showed that overexpression of CK2 is a recurrent event in both AML cell lines and patient samples (Fig. 4a, Supplementary Fig. S2). First, we confirmed that CK2 phosphorylation is indeed regulated by p38 in AML cells, as it is decreased after p38α or p38β knockdown (Fig. 4b). Next, we investigated whether p38β silencing or CK2 inhibition using CX-4945 affects SET phosphorylation, by using Phos-tag^TM^ SDS-PAGE. Inhibition of CK2 and silencing of p38β, but not p38α, substantially decreased the phosphorylated forms of SET in AML cells (Fig. 4c, Supplementary Fig. S8a). Interestingly, while both p38α and p38β can potentially regulate CK2 phosphorylation, only the inhibition of p38β affected SET phosphorylation and SET interaction with CK2.

In order to confirm that p38β regulates PP2A activity, we overexpressed p38β in HEK293T cells with the pEFM-link-p38β plasmid. The ectopic increment of p38β resulted in a significant increase in the phosphorylation of CK2, accompanied with a reduction of PP2A activity (Fig. 4d), suggesting that p38β is involved in CK2 activation and regulation of PP2A activity. To further demonstrate that CK2 is crucial in the decrease of PP2A activity produced by p38β overexpression, we inhibited CK2 by adding CX-4945 (3.75 µM) for 24 h. CK2 inhibition restored PP2A activity in cells overexpressing p38β (Fig. 4d) suggesting that CK2 is an intermediate in PP2A activity regulation by p38β. Additionally, to study whether CK2 has a direct effect on PP2A regulation, SET was silenced in both AML cell lines and then, the cells were treated with CX-4945 to inhibit CK2. Inhibition of CK2 in cells with reduced amount of SET had no effect in PP2A activity in the AML cells tested (Fig. 4e), suggesting that CK2-dependent inhibition of PP2A is through SET.

**Fig. 5:**
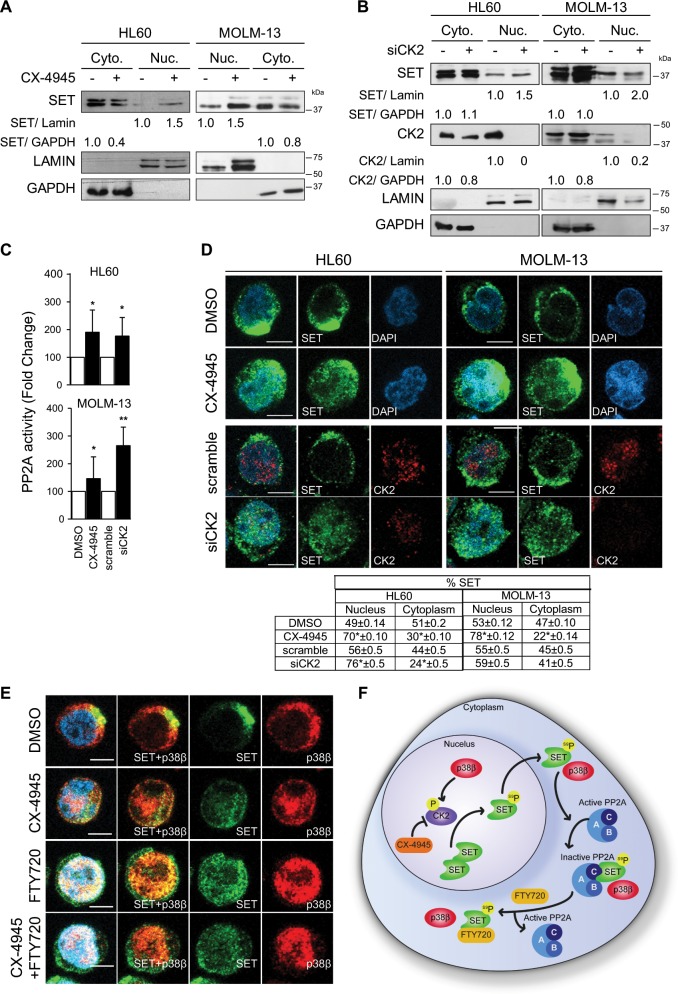
CK2 inhibition retains SET in the nucleus. **a** Nuclear (Nuc.) and cytoplasmic (Cyto.) protein isolated from HL60 and MOLM-13 cells treated with CX-4945 (5 µM, 24 h) and analyzed by western blot for SET localization. **b** HL60 and MOLM-13 cells treated with specific siRNA for CK2 (20 nM, 48 h). Nuclear (Nuc.) and cytoplasmic (Cyto.) proteins were isolated and analyzed by western blot for SET localization. **c** Measurement of PP2A activity by immunoprecipitation and phosphatase assay. The results are expressed as fold-change of the control, which are assigned a value of 1 and are mean values ± SEM. Experiments were performed in triplicate four times. *p < 0.05, **p < 0.01. **d** Immunofluorescence analysis of CK2 (red) and SET (green). Nuclei were stained with DAPI. Immunofluorescences were visualized by confocal microscopy. Scale bar represents 5 µm. Quantification table of green fluorescence (SET) in nucleus and cytoplasm. The results are expressed as mean values ± SEM. Experiments were performed in triplicate four times. *p < 0.05, **p < 0.01. **e** Immunofluorescence analysis of p38β (red) and SET (green). Nuclei were stained with DAPI. Immunofluorescences were visualized by confocal microscopy. Scale bar represents 5 µm. **f** p38β is able to activate CK2, which phosphorylates SET and, as consequence, facilities SET trafficking to the cytoplasm, contributing to PP2A inactivation in AML cells. Moreover, p38β binds to SET in the cytoplasm, contributing to its stability and leading to PP2A inactivation. Treatment with CX-4945 (CK2 inhibitor) retains SET in the nucleus, avoiding its phosphorylation. FTY720 treatment disrupts the SET-PP2A biding which remains in the cytoplasm.

Next, we treated AML cells with either CX-4945 or CK2 siRNA, and found that CK2 inhibition or downregulation resulted in increased nuclear and decreased cytoplasmic localization of SET (Fig. 5a, b, Supplementary Fig. S8b, c), which increased PP2A activity (Fig. 5c). These results were confirmed by immunofluorescence (Fig. 5d). Furthermore, nuclear SET retention was accompanied by enhanced p38β nuclear localization (Fig. 5e). Taken together, our results show that p38β-dependent activation of CK2 leads to SET phosphorylation, enhancing its cytoplasmic localization and consequently reducing PP2A activity (Fig. 5f).

### Inhibition of CK2 potentiates the anticancer activity of a PP2A-activating drug on AML cells

**Fig. 6:**
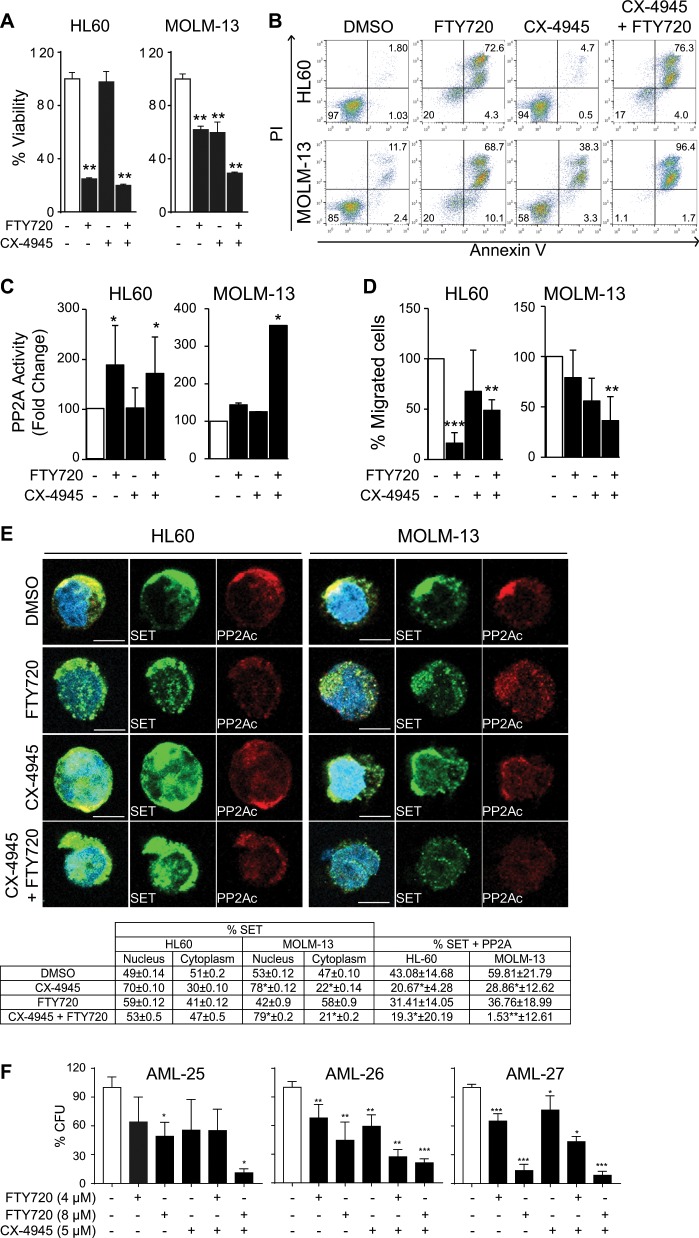
CX-4945 and FTY720 combination therapy induces apoptosis in AML cell lines and primary patient samples. HL60 and MOLM-13 cells pretreated for 4 h with CX-4945 (5 µM) and then treated for 24 h with FTY720 (5 µM). **a** Cell viability was measured by MTS analysis. The results are corrected by the DMSO control and are expressed as fold-change of the control, which are assigned a value of 1 and are mean values ± SEM. **b** FACS analysis of apoptosis in HL60 and MOLM-13 cell lines stained with propidium iodide (PI) and Annexin V. The percentages of viable and apoptotic cells are indicated. **c** PP2A activity analysis performed by immunoprecipitation and activity assay. **d** Migration of HL60 and MOLM-13 placed in the upper well of a 8.0 μM transwell plate in RPMI without FBS. The lower chamber contained RPMI supplemented with 10% FBS. Migration assay was performed for 3 h and then assessed for cell number using flow cytometry **e** Immunofluorescence analysis of SET (green) and PP2A (red) and quantification table of % colocalization between red fluorescence (PP2A) and green fluorescence (SET) and % of green fluorescence (SET) in nucleus and cytoplasm. Nuclei were stained with DAPI. Immunofluorescences were visualized by confocal microscopy. Scale bar represents 5 µm. Experiments were performed in triplicate four times. *p < 0.05, **p < 0.01. **f** AML patient samples were cultured in semisolid medium and treated with CX-4945 (5 µM) and FTY720 (4 and 8 µM), alone or in combination. Colony formation units (CFU) were counted 12 days after seeding. Graphs of counted CFU represented as percentage of CFU related to the control, which are assigned the total CFU (100%) and are mean values ± SEM.

We have shown that p38β contributes to the inactivation of PP2A in AML cells, which involves phosphorylation of SET by CK2. Therefore, we speculated that CK2 inhibition by CX-4945 could enhance the antileukemic effect of the PP2A-activating drug FTY720 that binds SET^19^. To test this idea, we first used immunofluorescence to analyze the time-course of SET nuclear retention after CK2 inhibition, which started at 4 h (Supplementary Fig. S9). Accordingly, AML cells were treated with CX-4945 for 4 h prior to FTY720 treatment. The combined treatment significantly decreased cell viability (Fig. 6a) and increased apoptosis (Fig. 6b) in AML cells, being more effective than either single treatment and having a synergistic effect (Supplementary Fig. 10a). These effects correlated with increased PP2A activity (Fig. 6c) and reduced cell migration ability (Fig. 6d). Immunofluorescence analysis showed that the combined treatment retained SET in the nucleus together with p38β, and disrupted the SET-PP2Ac interaction (Fig. 6e), supporting the mechanism proposed. We also evaluated the combined treatment in AML primary patient samples. According to availability, we treated patient-derived PB-MC samples (Supplementary Table S1) with FTY720, CX-4945 or both, and then performed MTS assays. When there was enough sample, we also tested the colony formation ability. We found decreased viability in 64% of the samples treated with FTY720 (15/24), and in 90% of the samples treated with CX-4945 (10/11). Importantly, all five AML patient samples that were treated with both drugs showed a significant decrease in cell viability compared to either treatment alone (a representative case shown in Supplementary Fig. S10b). Colony formation ability was also reduced in the three AML patient samples treated with both drugs that were grown in semisolid medium (Fig. 6f, Supplementary Fig. S10c).

**Fig. 7:**
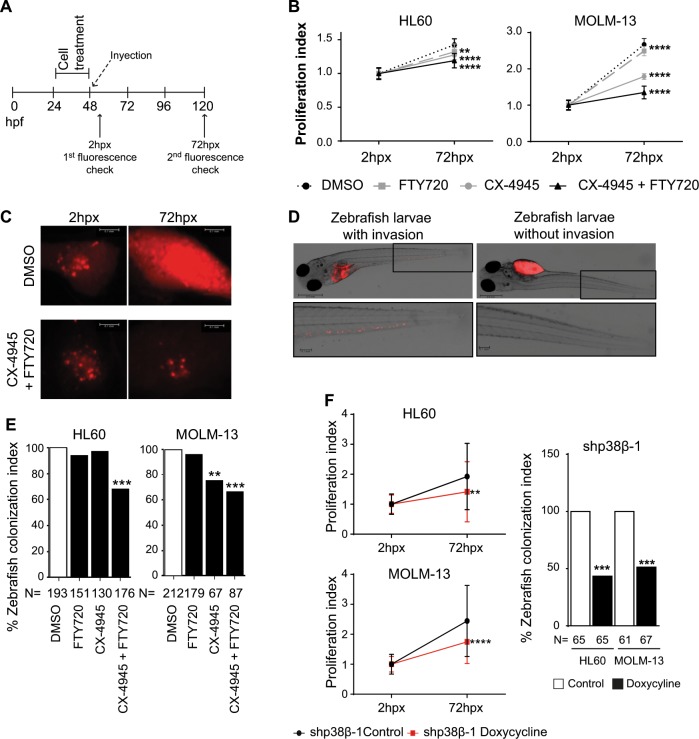
CX-4945 and FTY720 combination therapy induces AML cell death in zebrafish xenograft models. In vivo proliferation and invasive potential of HL60 and MOLM-13 cells upon treatment with FTY720, CX-4945 or the combination of both compounds were analyzed in a xenograft zebrafish model. **a** Timing scheme for the xenografts of zebrafish embryos. **b** Measurement of proliferation index performed as fluorescence intensity medium value* RF pixel, demonstrating cell proliferation of treated cells in the xenograft model. **c** Representative pictures of Tumor growth of HL60 treated cells in zebrafish embryos 2 hpx (reference fluorescence) and 72hpx. Scale bars represent 0.1 mm. **d** Representative pictures of zebrafish embryos injected with MOLM-13 cells and treated with DMSO or combination of CX-4945 (1 µM) and FTY720 (1 µM), which show the cells that migrated to the tail after the treatment mentioned. Magnified pictures on the bottom show the invasion of cells in the tail. Scale bars from whole zebrafish picture represent 0.5 mm and from zoom 0.1 mm. **e** Quantification of the invasive potential of the injected cells upon drug treatment. Quantification performed as colonization index: count of zebrafish embryos with invasion of cells migrating outside the yolk sac referred to the control embryos (injection of DMSO treated cells). Hpx: hours post-xenograft. **p < 0.01, ***p < 0.001 vs. DMSO treated cells. **f** In vivo proliferation and invasive potential of HL60 and MOLM-13 cells upon infection with shp38β pINDUCER11 treated with and without doxycycline in a xenograft zebrafish model measured at 72hpx. **p < 0.01, ***p < 0.001 vs. control cells.

Finally, we validated the proposed mechanism in a zebrafish xenograft model, a robust animal system to test tumor cell behavior and drug response^34–36^. AML cells were evaluated for in vivo proliferation and invasion potential in zebrafish embryos upon treatment with FTY720, CX-4945 or their combination, following the scheme in Fig. 7a. Embryos were analyzed 2 h post-xenograft (hpx) to confirm proper injection, and 72hpx for proliferation and invasion. The combined treatment significantly decreased the proliferation index compared to both single treatments and the control (Fig. 7b), as well as the tumor growth area in zebrafish embryos (Fig. 7c). We also studied the colonization potential of treated AML cells by analyzing the zebrafish larvae with invasion in the tail, as illustrated in Fig. 7d. Quantifications demonstrated that treatment with CX-4945 and FTY720 significantly reduced zebrafish larvae with AML cell tail invasion (Fig. 7e). To corroborate the importance of p38β in our model, we injected zebrafish embryos with AML cells expressing two different doxycycline inducible p38β shRNAs (Fig. 7a). We found that p38β silencing decreased the proliferation and colonization index of AML cells in zebrafish embryos 72hpx (Fig. 7f, Supplementary Fig. S11), supporting the functional importance of p38β overexpression in AML cells. Taken together, our results combining FTY720 with CK2 inhibitors or using p38β shRNAs support the proposed new mechanism that regulates AML cell viability and migration.

## Discussion

Here, we investigated the post-transcriptional regulation of the SET oncoprotein, establishing that p38β overexpression is a common event in AML that leads to PP2A inactivation through its endogenous inhibitor SET. Furthermore, we provide evidence that p38β, but not the closely related p38α family member, controls the phosphorylation of SET by CK2, facilitating SET shuttling from the nucleus to the cytoplasm. Besides, p38β also acts as a SET stabilizing protein, facilitating PP2A inactivation. We describe a novel molecular pathway of leukemogenesis with therapeutic potential in AML patients that show SET-dependent PP2A inactivation, a subgroup with poor prognosis that represent ~30% of all AML cases. PP2A is a tumor suppressor, which regulates most of the kinase-driven intracellular signaling pathways. Thus, by targeting SET, this approach allows reactivating PP2A indirectly, avoiding toxicity issues related to the direct activation of this complex holoenzyme.

Mitogen-activated protein kinase (MAPK) cascades are important signaling pathways used by eukaryotic cells to transduce extracellular signals. Using chemical inhibitors of several MAPKs, we found that only p38 inhibitors were able to increase PP2A activity by decreasing SET protein, suggesting post-transcriptional regulation of SET. The p38 family is involved in many cellular processes, and plays a key role in the stress response^37^. Although p38α and p38β share 70% in amino acid sequence homology, they have different functions^37,38^ and differential regulatory mechanisms^39^. Nevertheless, the high expression of p38α in most tissues, together with the results using knockout mice deficient in p38α or p38β, suggests that p38α is the dominant form, although functional redundancy has been reported^40^. As a consequence, most studies have focused on p38α, and little is known about p38β^37^. In this regard, we have elucidated an important role of p38β in AML that has not been previously suggested. p38β is widely expressed, but usually at low levels in most tissues^37^, and it is not detected in monocytes, macrophages, or neutrophils^41^. We found that upregulation of p38β, but not p38α, is a common event in AML cases that contributes to the SET-dependent inactivation of PP2A in AML, pointing to a relevant role of p38β in this aggressive disease.

It has been reported that PP2A can regulate p38 signaling pathway, and that PP2A and p38 form complexes in the cytoplasm in basal conditions; in fact, p38 may act as scaffold protein for NMP and PP2A^42–45^. However, the nature of their connection varies depending on the context. Upon TNF-induced stress conditions in endothelium-derived cell lines, p38 positively regulates PP2A activity^42^, whereas under hypoxia and survival conditions in colorectal cancer cell lines, PP2A negatively regulates p38MAPK activity^44^. Here, we show for the first time that p38β controls PP2A activity through the regulation of its endogenous inhibitor SET.

Pyridinyl-imidazole inhibitors have allowed the identification of many functions regulated by p38 beyond the stress response. However, these compounds do not permit us to distinguish functions mediated by p38β from those regulated by p38α. Therefore, we used RNA interference to decipher the specific role of p38β in AML cells. Our immunoprecipitation and immunofluorescence analysis support the notion that p38β interacts with SET in AML cells, and knockdown of p38β, but not p38α, decreases SET protein levels and enhances PP2A activity. Moreover, we found that p38β binding stabilizes the SET protein in the cytoplasm, demonstrating a new role for p38β. We had previously demonstrated that SETBP1 binds to and stabilizes SET, facilitating PP2A inhibition^25^, and this result has been confirmed in other reports^46,47^. Here we further characterize this mechanism by showing that p38β co-localizes with SET, SETBP1, and PP2A, regulating PP2A activity in AML cells. Additional studies will be needed to elucidate how the interplay among these proteins regulates SET stability.

SET is mostly located in the nucleus where it regulates DNA replication, chromatin remodeling, gene transcription^20^, DNA repair^21^, migration^22^, and cell-cycle progression^23^. Here, we report a robust accumulation of SET into the cytoplasm of primary and patient-derived AML cells. Several studies in Alzheimer’s disease show that CK2 phosphorylates SET on Ser9, in the nuclear localization signal, which is key for SET cytoplasmic localization and inhibition of PP2A, leading to tau hyperphosphorylation^30,31,48^. Here we demonstrate in AML cells that p38β is involved in SET trafficking to the cytosol and PP2A inactivation through the activation of CK2, and that silencing of p38β but not p38α decreases CK2-dependent phosphorylation of SET. Moreover, overexpression of p38β decreased PP2A activity in a CK2-dependent manner. Consistent with these findings, pharmacological inhibition or silencing of CK2 increased the nuclear localization of SET, as well as PP2A activity, without altering total SET protein levels. Interestingly, in the absence of SET, inhibition of CK2 has no effect in PP2A activity, supporting the new mechanism described here. It should be noted that CK2 overexpression has been associated with poor prognosis in AML patients with normal karyotype^32,49^. Thus, our results support a model in which p38β overexpression activates CK2, which in turn phosphorylates SET, facilitating its trafficking to the cytoplasm where it inactivates PP2A. Therefore, our study identifies a novel p38β-CK2-SET signaling pathway in leukemogenesis that mediates PP2A inactivation. Interestingly, the same pathway would be probably activated in AML cases with CK2 overexpression, opening new insights into the role of CK2 in AML.

We validated the importance of this new pathway by showing that the combination of CX-4945, which inhibits CK2 allowing nuclear SET retention, and FTY720, which disrupts SET-PP2A binding, is more effective in decreasing viability and inducing apoptosis of AML cells from patients than either single treatment. In vivo studies using zebrafish xenografts as a preclinical model^34^ supported the importance of p38β in AML cells, and confirmed that the combination of CX-4945 and FTY720 is more effective than either treatment alone at reducing tumor growth, as well as impairing cell migration and invasion. Consistent with our results, SET phosphorylation would allow its interaction with the GTPase Rac1 and cytoplasm localization, where SET inactivates PP2A. In fact, it has been reported that Rac1 stimulated signaling required for efficient cell migration involves SET-mediated inhibition of PP2A, and that cytoplasmic targeting of SET inhibits Rac1-induced cell spreading and migration^22^. Taken together, our in vivo results confirm the value of targeting multiple components of the same pathway, and support the use of zebrafish xenografts to predict drug sensitivity for personalized treatments in AML cases.

In conclusion, we have identified a new role of p38β MAPK and CK2 in AML leukemogenesis. We show that p38β overexpression is a recurrent event in AML cases that contributes to PP2A inactivation by regulating the SET oncoprotein through two mechanisms: (i) p38β controls CK2-mediated phosphorylation of SET facilitating its cytoplasmic localization, and (ii) p38β binds to and stabilizes SET in the cytoplasm. Furthermore, we provide in vivo evidence of this mechanism by targeting the same pathway at different levels. We show that a combination therapy using the CK2 inhibitor CX-4945, which retains SET in the nucleus, and FTY720, which disrupts the SET-PP2A binding in the cytoplasm, re-activates PP2A, reducing the viability of AML cells. Our results therefore provide the rationale for using a combination of PP2A-activating drugs and CK2 inhibitors as a novel therapeutic option for treating a subgroup of 30% AML cases characterized by SET-dependent PP2A inactivation.

## Supplementary information

Supplemental Material

Reporting Checklist
