# Supplementary material for: A new regulatory mechanism of protein phosphatase 2A activity via SET in acute myeloid leukemia
Source: Blood Cancer J. 2020 Jan 8;10(1):3. doi: 10.1038/s41408-019-0270-0 (PMC6949222; doi:10.1038/s41408-019-0270-0)
Supplement: Supplementary file 1 — Supplemental Material [file 41408_2019_270_MOESM1_ESM.pdf]

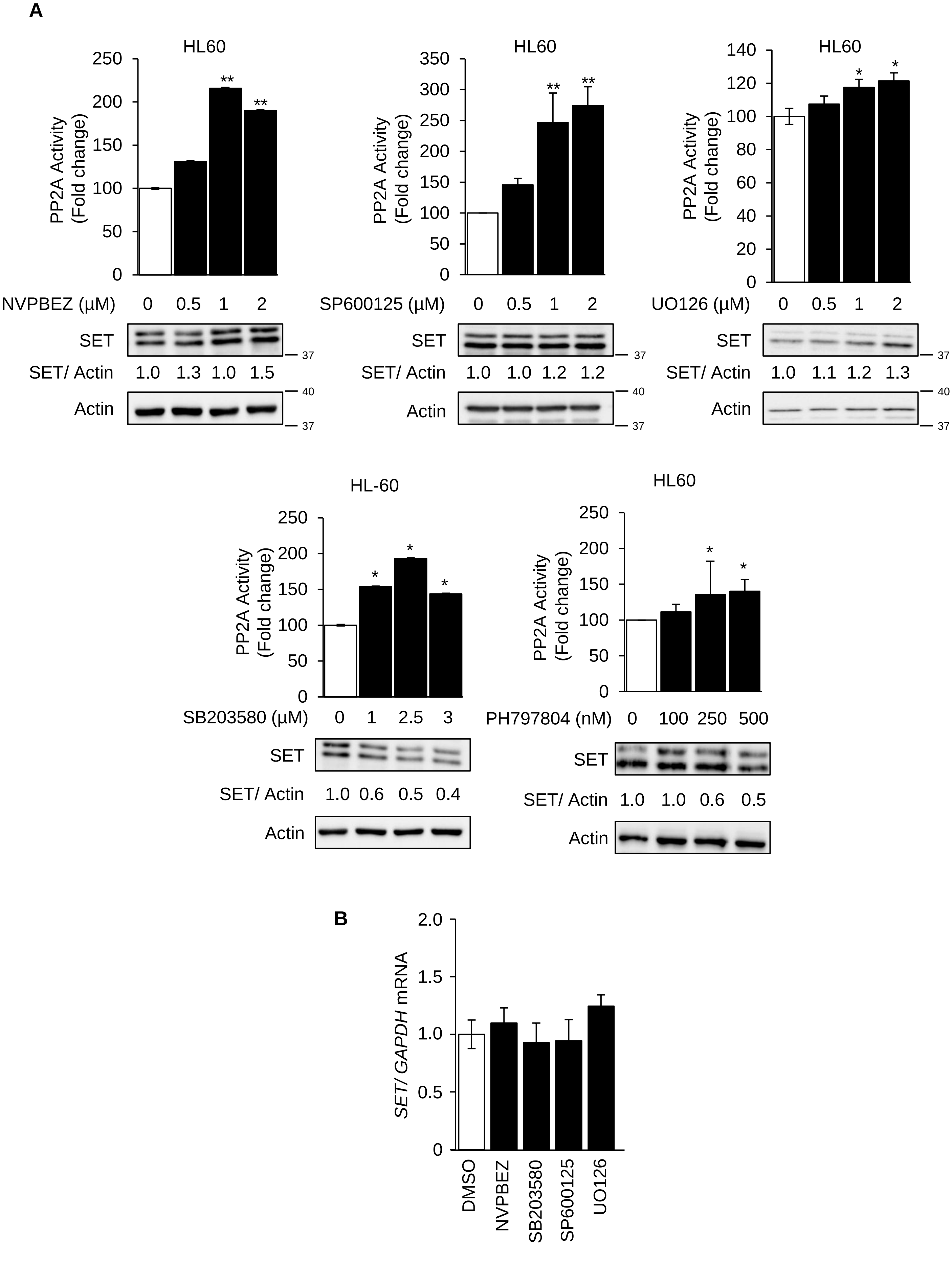
A
HL60
PP2A Activity (Fold change)
250
200
150
100
50
0
**
**
NVPBEZ (µM) 0 0.5 1 2
SET
37
SET/ Actin 1.0 1.3 1.0 1.5
40
Actin
37
HL60
PP2A Activity (Fold change)
350
300
250
200
150
100
50
0
**
**
SP600125 (µM) 0 0.5 1 2
SET
37
SET/ Actin 1.0 1.0 1.2 1.2
40
Actin
37
HL60
PP2A Activity (Fold change)
140
120
100
80
60
40
20
0
*
*
UO126 (µM) 0 0.5 1 2
SET
37
SET/ Actin 1.0 1.1 1.2 1.3
40
Actin
37
HL-60
PP2A Activity (Fold change)
250
200
150
100
50
0
*
*
*
SB203580 (µM) 0 1 2.5 3
SET
SET/ Actin 1.0 0.6 0.5 0.4
Actin
HL60
PP2A Activity (Fold change)
250
200
150
100
50
0
*
*
PH797804 (nM) 0 100 250 500
SET
SET/ Actin 1.0 1.0 0.6 0.5
Actin
B
SET/ GAPDH mRNA
2.0
1.5
1.0
0.5
0
DMSO
NVPBEZ
SB203580
SP600125
UO126

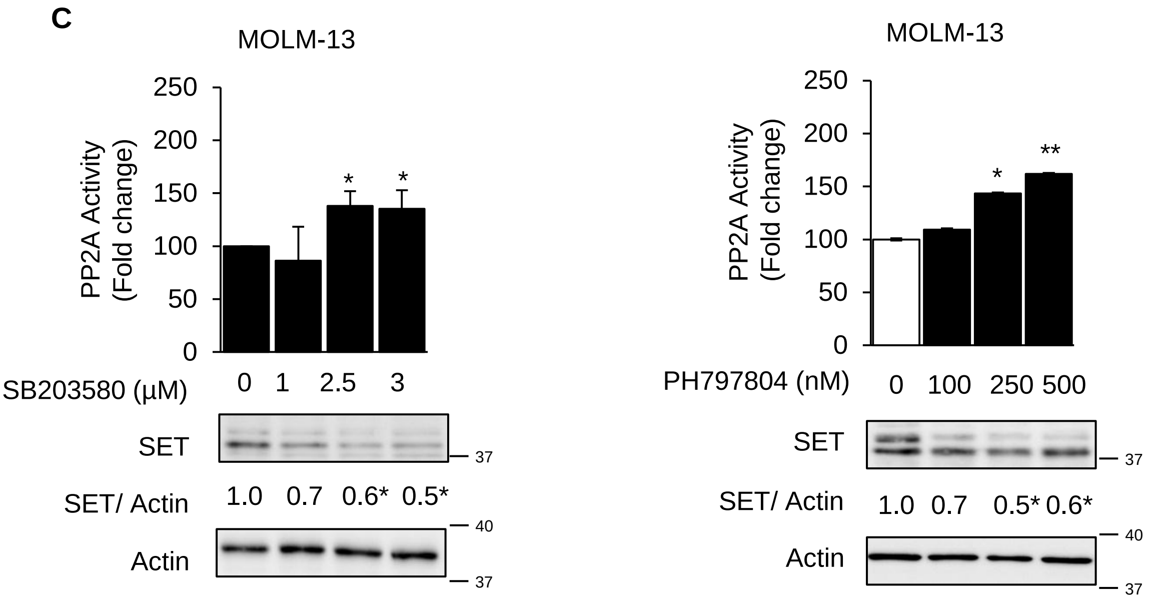

Figure S1. p38MAPK inhibition decreases SET oncoprotein, increasing PP2A activity in AML cells. HL60 cell line was treated with NVPBEZ (0.5, 1 and 2 µM, PI3K inhibitor), SB203580 (1, 2.5 and 3 µM, p38MAPK Inhibitor), PH797904 (100, 250 and 500 nM, p38MAPK inhibitor), SP600125 (0.5, 1 and 2 µM, JNK inhibitor) and UO126 (0.5, 1 and 2 µM, ERK inhibitor) for 24h. **(A)** Protein expression were analyzed by western blot. Measurement of PP2A activity by immunoprecipitation and phosphatase assay. **(B)** Quantitative RT-PCR analysis of the SET gene after treatment with NVPBEZ (1µM, PI3K inhibitor), SB203580 (2.5µM, p38 Inhibitor), PH797904 (250nM, p38MAPK inhibitor), SP600125 (1µM, JNK inhibitor) and UO126 (1µM, ERK inhibitor) for 24h. **(C)** MOLM-13 cell line was treated with SB203580 (1, 2.5 and 3 µM, p38MAPK Inhibitor) and PH797904 (100, 250 and 500 nM, p38MAPK inhibitor) for 24h. Protein expression were analyzed by western blot. Measurement of PP2A activity by immunoprecipitation and phosphatase assay. The results are corrected by the specific loading control and are expressed as fold-change of the control, which are assigned a value of 1 and are mean values±SEM. Experiments were performed in triplicate four times. *p<0.05.

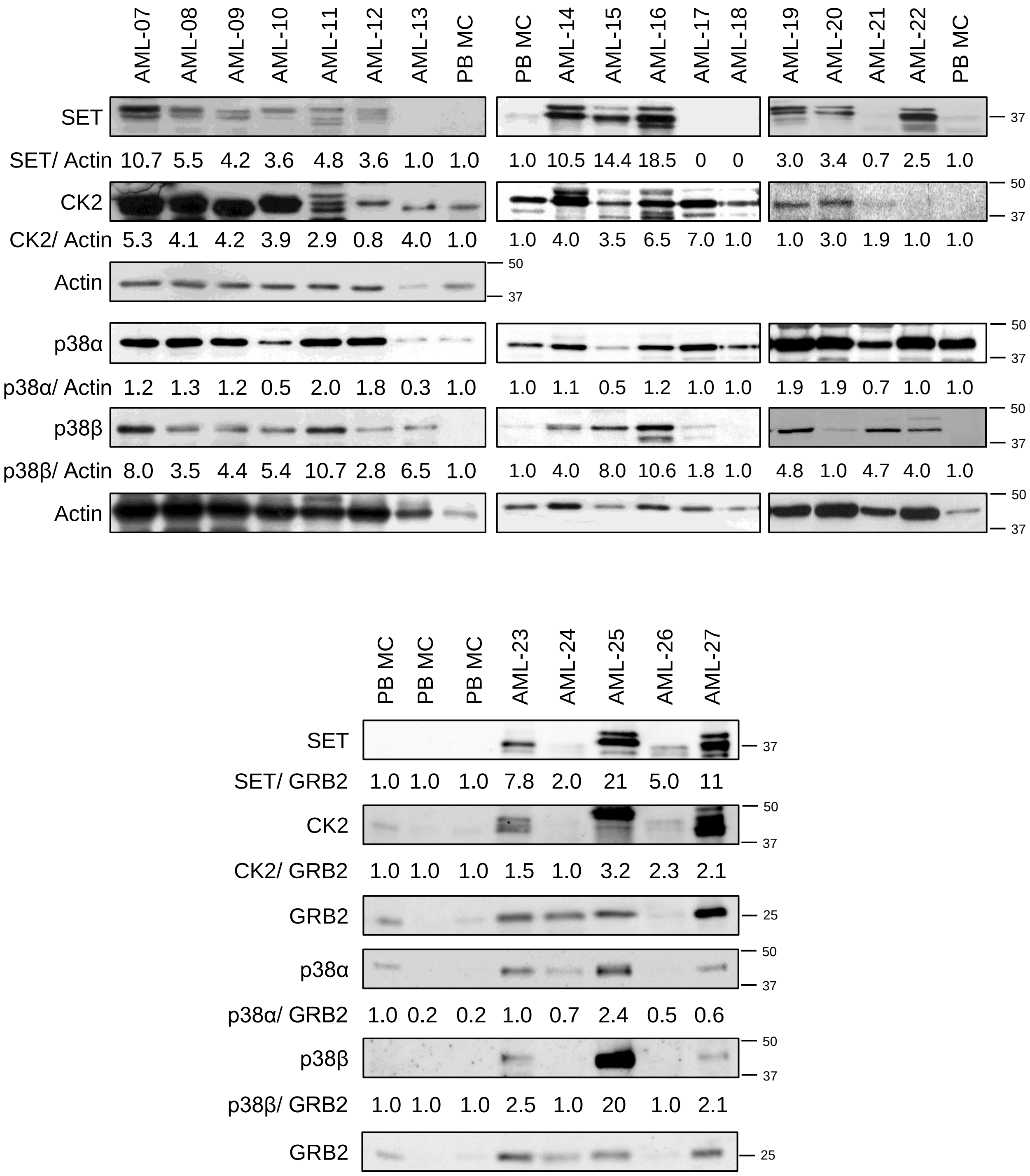

Figure S2. p38β, CK2 and SET proteins are overexpressed in AML patient samples. Western blot analysis of SET, p38α, p38β and CK2 from AML patient samples compared to peripheral blood mononuclear cells (PB MC).

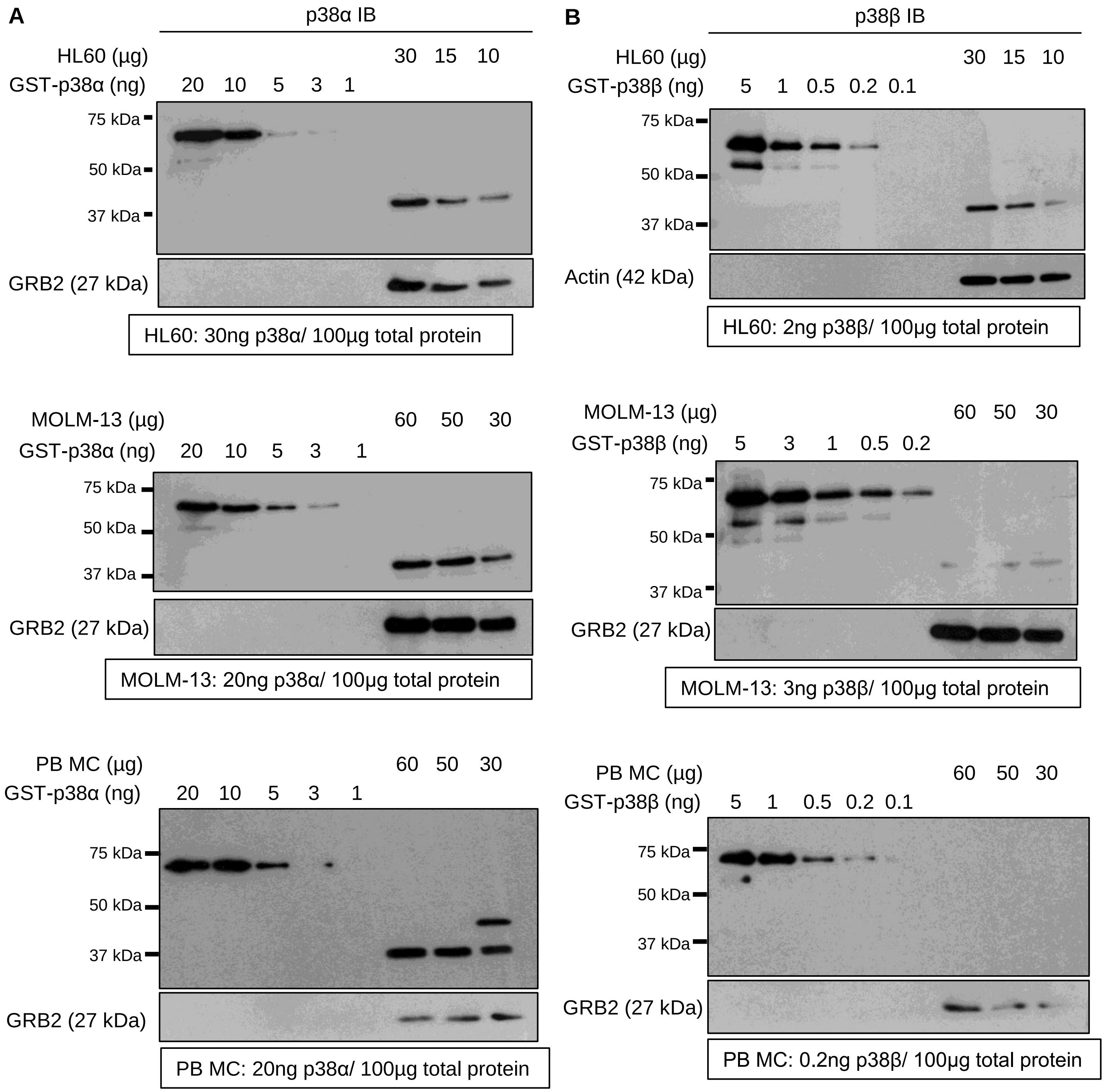

Figure S3. Expression of p38α and p38β in HL60, MOLM-13 and peripheral blood. Quantitative western blot using known decreasing concentrations of GST-p38α and GST-p38β compared to increasing concentration of cell lysates from HL60, MOLM-13 and peripheral blood mononuclear cells (PB MC). Western blot with specific antibodies and subsequent quantification was performed. **(A)** p38α is expressed in a similar way in HL60, MOLM-13 and peripheral blood. **(B)** p38β is expressed in HL60 and MOLM-13 cell lines and not in PB MC.

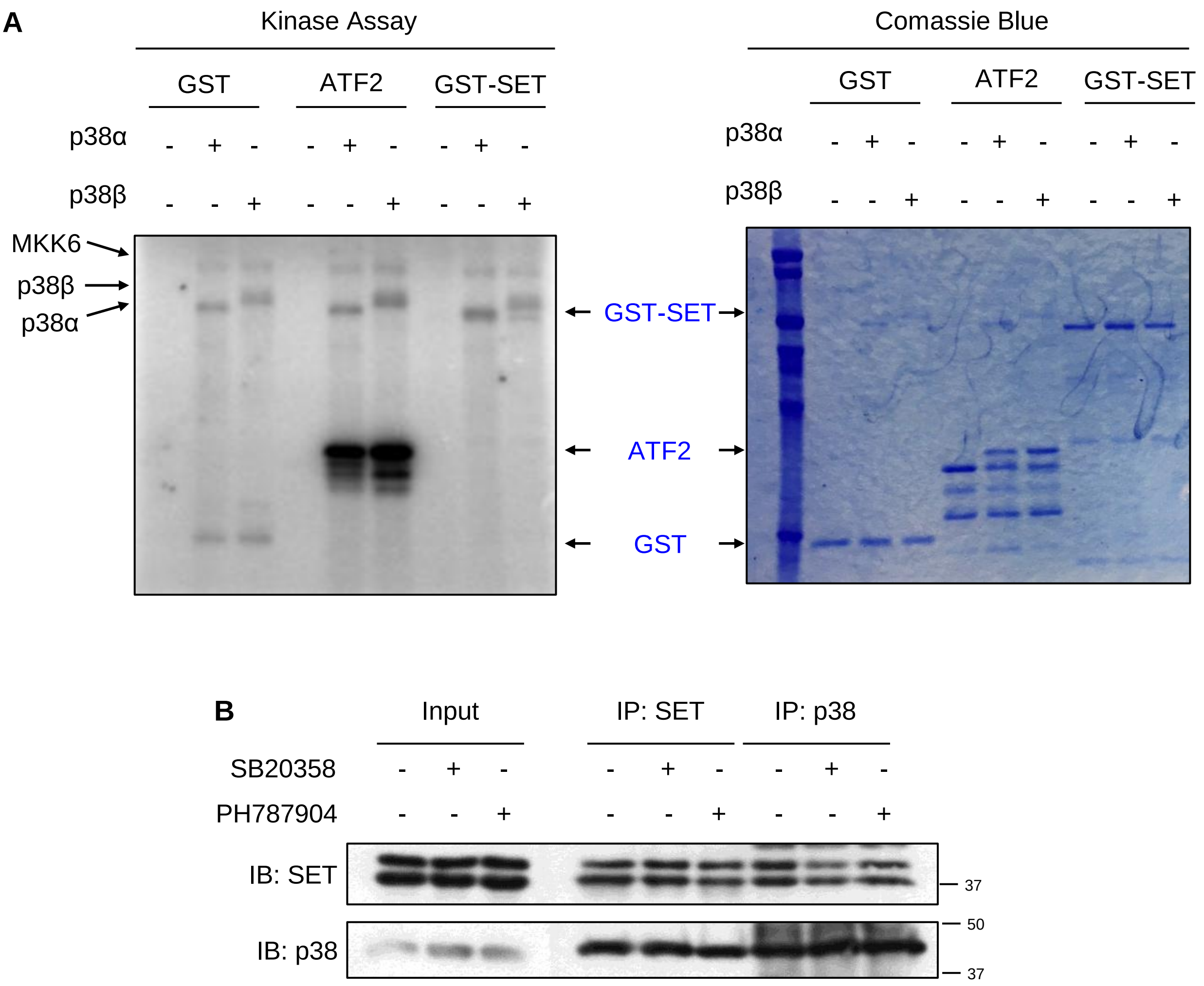

Figure S4. p38MAPK does not phosphorylate directly SET and p38MAPK does not need to be active to bind to SET. (A) *In vitro* kinase assay using p38α or p38β and either GST (negative control), ATF2 (positive control) or GST-SET in the presence of ^{32}P. p38α and p38β were activated with MKK6. On the left autoradiography of the kinase assay; on the right comassie blue staining of the same gel. **(B)** HL60 were treated with p38MAPK inhibitors SB203580 (2.5µM, 24h) and PH787904 (1µM, 24h) and then, immunoprecipitation of SET and p38MAPK with specific antibodies was performed, followed by western blot.

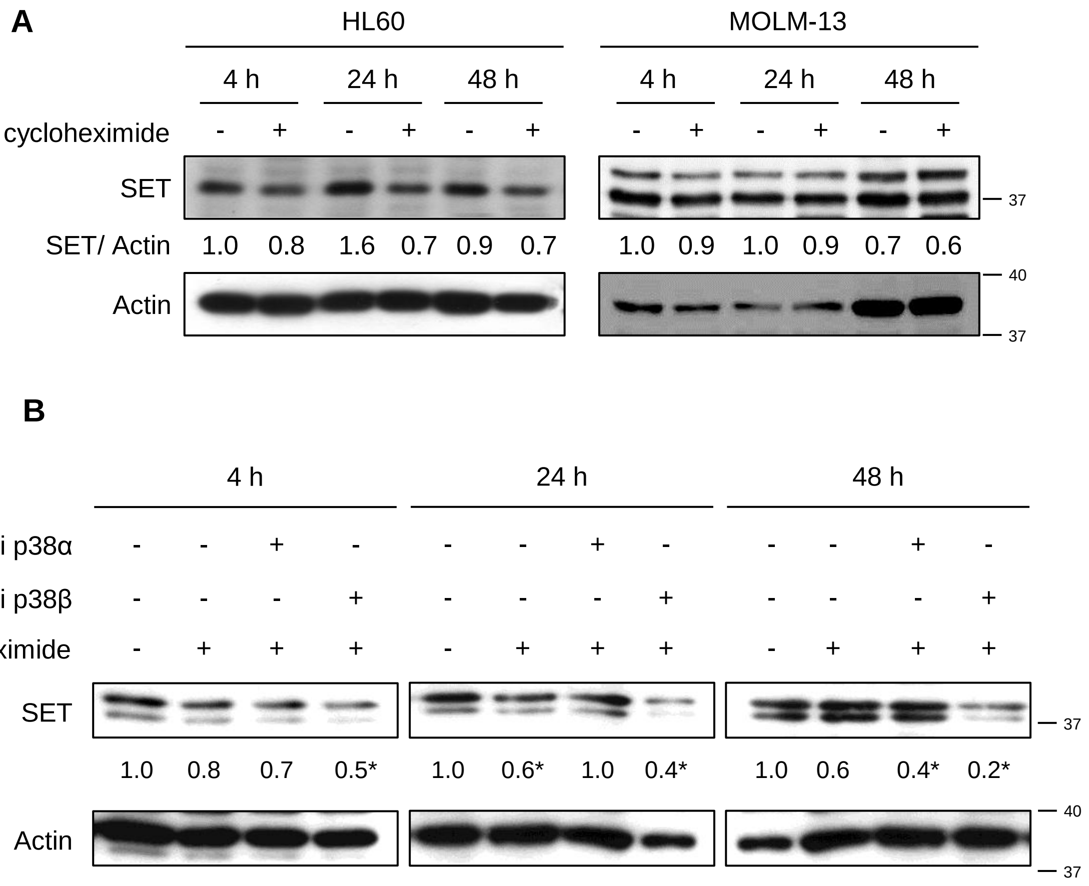

Figure S5. Silencing of p38β decreases SET stability. (A) HL60 and MOLM-13 cell lines were treated with 5µM of cycloheximide (CHX) for 4h, 24h and 48h to inhibit total protein synthesis, and SET protein was analyzed by western blot. **(B)** Silencing of p38α and p38β with specific siRNA (50nM for 48h), in either the presence or absence of CHX (5µM, for 4, 24 or 48 h). Total protein was analyzed by western blot. The results are corrected by the specific loading control and are expressed as fold-change of the control, which are assigned a value of 100 and are mean values±SEM. Experiments were performed in triplicate four times. *p<0.05, ** p<0.01.

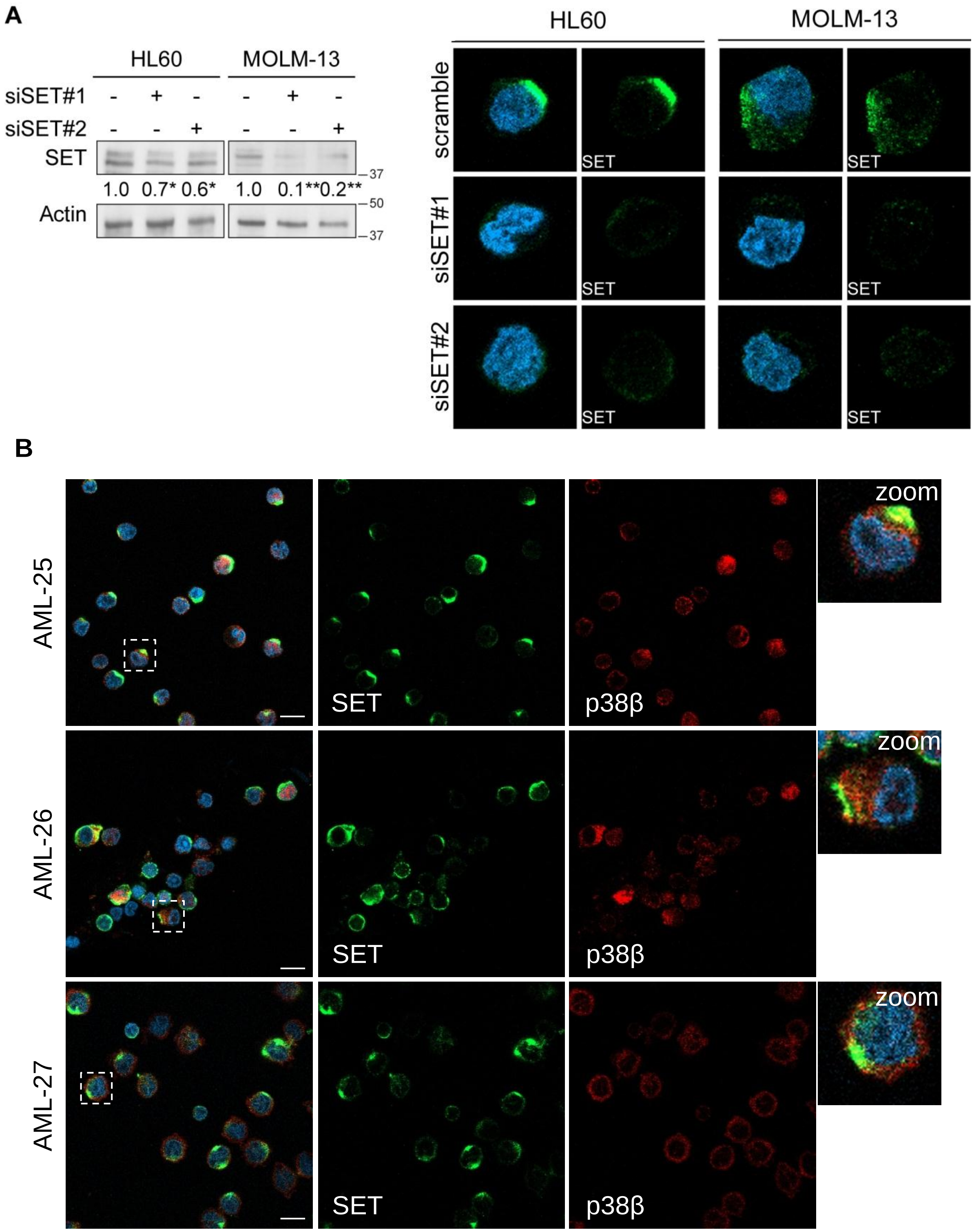

Figure S6. SET co-localizes with p38β in AML patient samples. (A) HL60 and MOLM-13 cells were silenced for SET with specific siRNAs (50nM for 72h). Total protein was analyzed by western blot. Immunofluorescence analysis of SET (green) in AML cells silenced for SET was performed as a control for SET antibody specificity. **(B)** Immunofluorescence analysis of SET (green) and p38β (red) co-localization in AML patient samples. Immunofluorescences were visualized by confocal microscopy. Scale bar represents 5 µm.

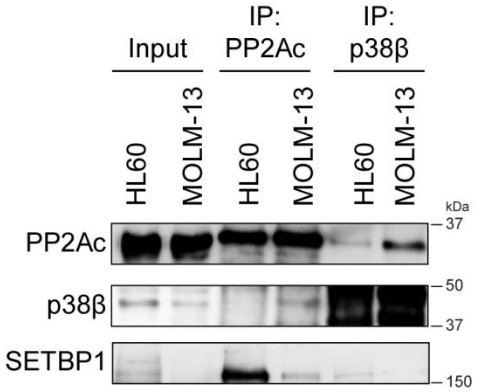

Figure S7. SETBP1 immunoprecipitates along with PP2A2c and p38β. Immunoprecipitation of PP2Ac or p38β and immunoblot of either PP2Ac, p38β or SETBP1 in HL60 and MOLM-13 cells Experiments were performed in triplicate four times

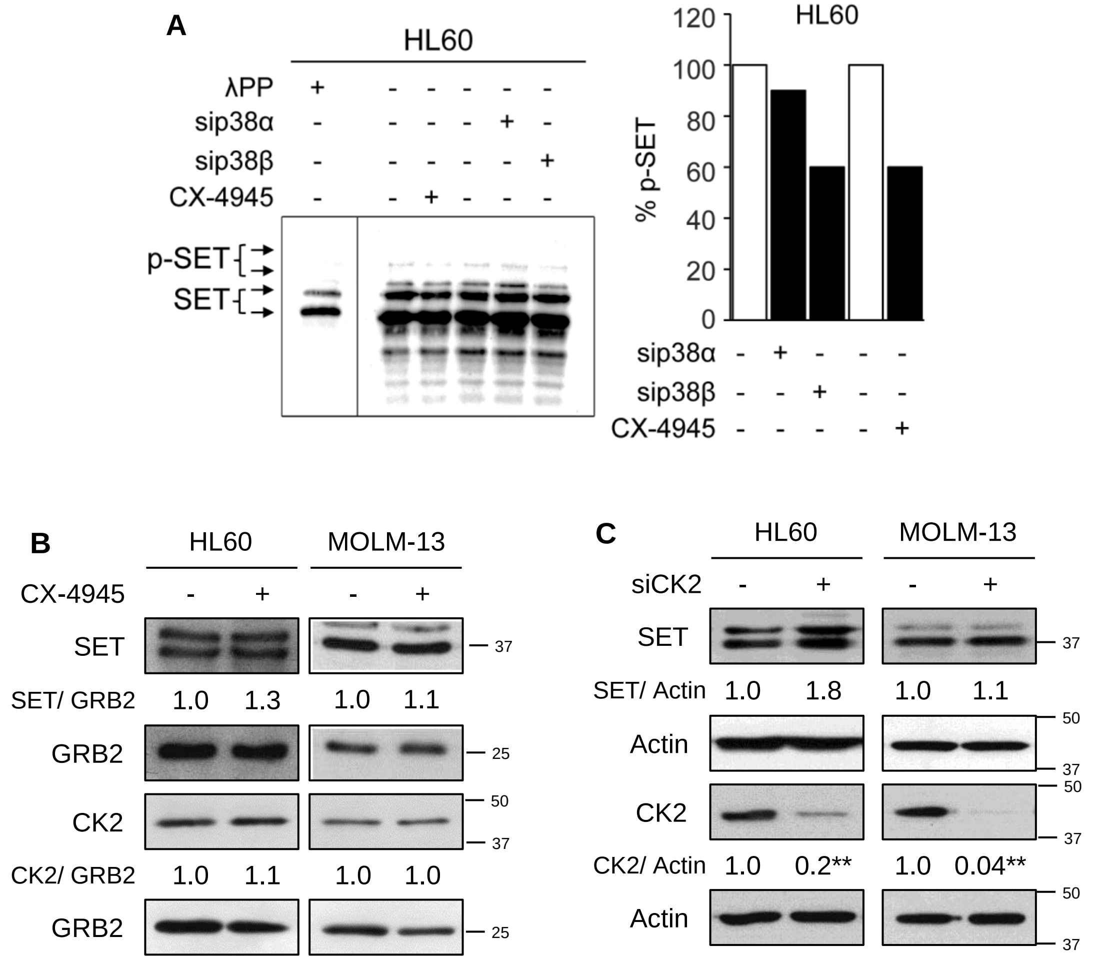

Figure S8. CK2 inhibition does not alter SET total protein (A) HL60 cells treated with either siRNA for silencing p38α and p38β (50nM for 48h) or with the CK2 inhibitor CX-4945 (5μM, 24h) and analyzed for phospho-SET in a SDS-page with Phos-TagTM. A control sample treated with λPP (100 units, 1h) was used as control. **(B)** HL60 and MOLM-13 cell lines were treated with the CK2 inhibitor CX-4940 (5μM, 24h). Total protein expression of SET and CK2 was analyzed by western blot. **(C)** HL60 and MOLM-13 cells were treated with CK2 specific siRNA (20nM, 48h). Total protein expression of SET and CK2 was analyzed by western blot. The results are corrected by the specific loading control and are expressed as fold-change of the control, which are assigned a value of 1 and are mean values±SEM. Experiments were performed in triplicate four times. ** *p*<0.01. The results are corrected by the specific loading control and are expressed as fold-change of the control, which are assigned a value of 1 and are mean values±SEM. Experiments were performed in triplicate four times. *p<0.05 **p<0.01.

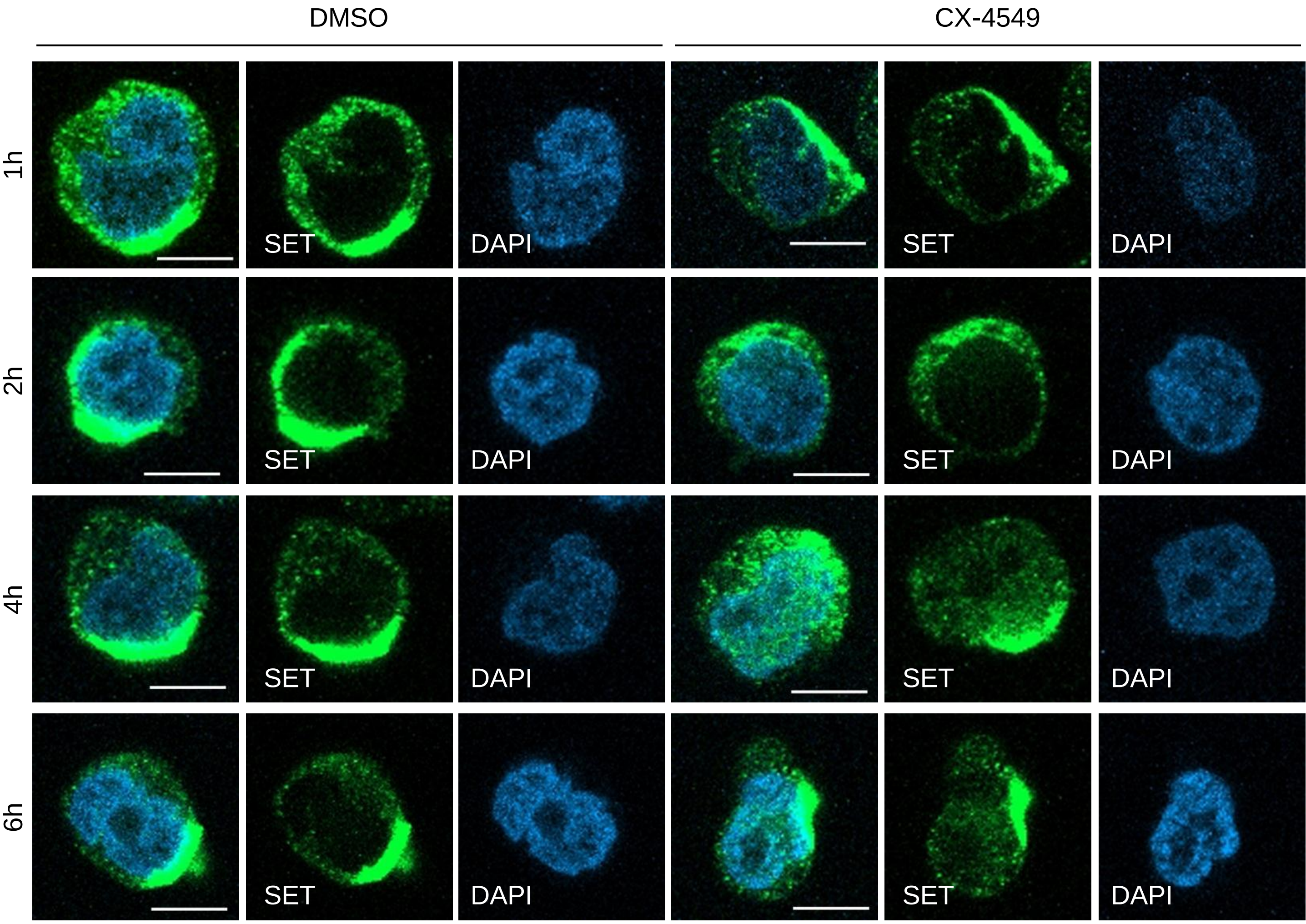

Figure S9. Inhibition of CK2 increased SET in the nucleus 4 h after treatment. Time course analysis of HL-60 cells treated with CX-4945 (CK2 inhibitor, 5µM) from 1h up to 6h. Immunofluorescence analysis of SET (green). Nuclei were stained with DAPI (blue). Immunofluorescences were visualized by confocal microscopy. Scale bar represents 5µm.

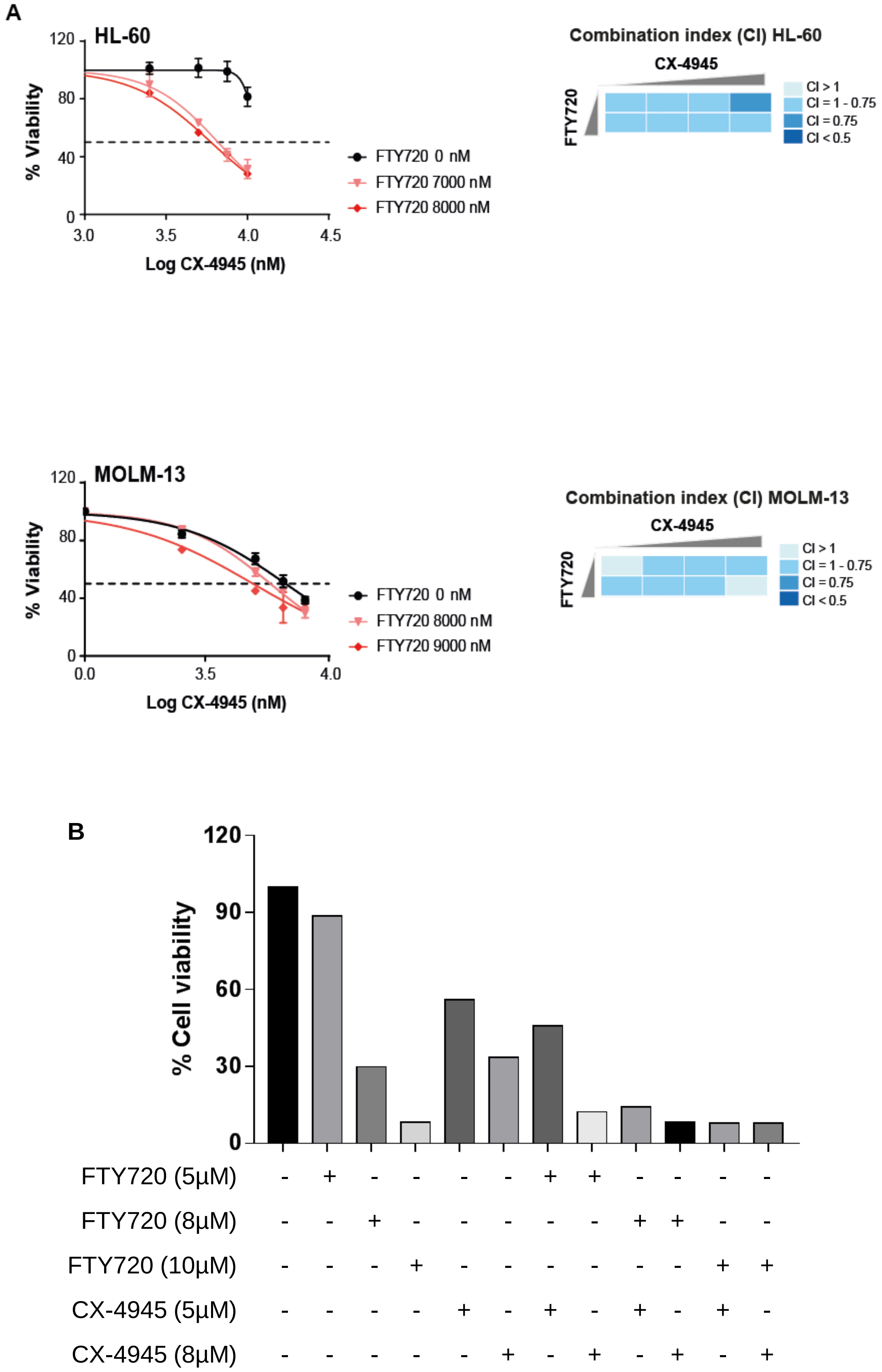
A
HL-60
% Viability
120
80
40
0
3.0
3.5
4.0
4.5
Log CX-4945 (nM)
FTY720 0 nM
FTY720 7000 nM
FTY720 8000 nM
Combination index (CI) HL-60
CX-4945
FTY720
CI > 1
CI = 1 - 0.75
CI = 0.75
CI < 0.5
MOLM-13
% Viability
120
80
40
0
0.0
3.5
4.0
Log CX-4945 (nM)
FTY720 0 nM
FTY720 8000 nM
FTY720 9000 nM
Combination index (CI) MOLM-13
CX-4945
FTY720
CI > 1
CI = 1 - 0.75
CI = 0.75
CI < 0.5
B
% Cell viability
120
90
60
30
0
FTY720 (5µM) - + - - - - + + - - - -
FTY720 (8µM) - - + - - - - - + + - -
FTY720 (10µM) - - - + - - - - - - + +
CX-4945 (5µM) - - - - + - + - + - + -
CX-4945 (8µM) - - - - - + - + - + - +

C

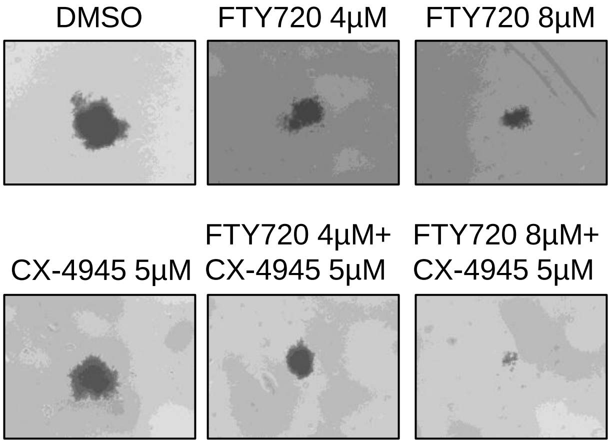

Figure S10. Combination therapy of CX-4945 and FTY720 decreases viability and colony formation in primary AML patient samples representative cases. (A) The GI50 (growth inhibition of 50%) of each compound was calculated using the data from the MTS assay in a non-linear regression model with the GraphPad Prism v7 software. To study the effect between these drugs, the GI50 of CX-4945 with different concentrations of FTY720 and vice versa were calculated. Combination Index (CI) values were calculated using the CompuSyn software (ref). The combined treatment was considered synergistic when the CI value was below 1. combination of CX-4945 and FTY720 resulted in a significant dose-dependent reduction of single treatment GI50 values in HL-60 and MOLM-13 cells. Furthermore, the calculated combination index values for the combined treatments were below 1 **(B)** Viability assay performed in AML-23 patient sample treated with CX-4945 (5 and 8 µM) and FTY720 (5, 8 and 10 µM) alone or in combination. Cell viability was measured by MTS analysis. The results are corrected by the DMSO control and are expressed as fold-change of the control, which are assigned a value of 1 and are mean values±SEM. **(C)** AML-26 patient sample cultured in semi-solid medium and treated with CX-4945 (5µM) and FTY720 (4 and 8 µM), alone or in combination. Colony formation units (CFU) were counted 12 days after seeding. Representative pictures of CFU are shown.

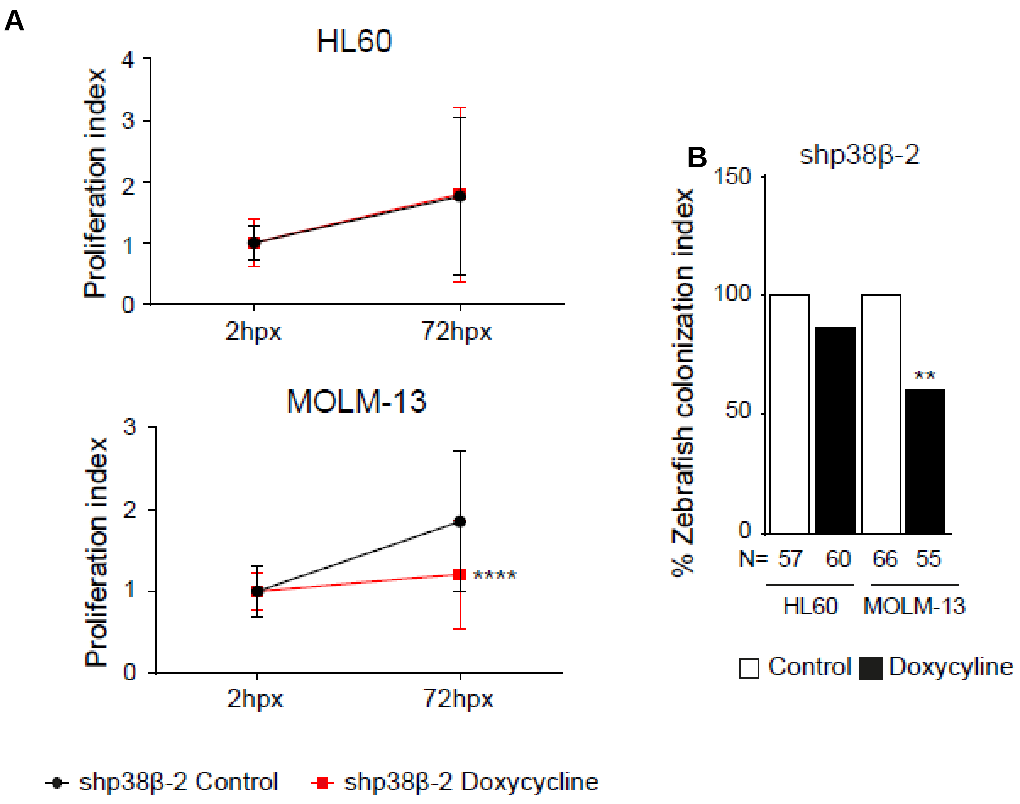

Figure S11. p38β knockdown in HL60 and MOLM-13 cells decreases their proliferation and migration in a zebrafish xenograft model. *In vivo* proliferation and invasive potential of HL60 and MOLM-13 cells upon infection with doxycycline shp38β vector and treatment with or without doxycycline were analyzed in a xenograft zebrafish model. **(A)** Measurement of proliferation index performed as fluorescence intensity medium value* RF pixel; demonstrating cell proliferation of treated cells in the xenograft model. Two way ANOVA (Tukey's multiple comparisons test) done as statistic analysis. **(B)** Quantification of the invasive potential of the injected cells upon doxycycline treatment. Quantification performed as colonization index: count of zebrafish embryos with invasion of cells migrating outside the yolk sac referred to the control embryos. Statistical analysis done was Chi^2. Hpx: hours post xenograft. ** p<0.01, ***p<0.001 *vs*. untreated cells.

Table S1. Characteristics of the 23 AML patient samples. Protein analysis was measured by western blot. High expression was considered as at least the double amount versus the control. ND: no data.

Case	Age	Gender	WHO 2016	Karyotype	Protein over expression			
					SET	CK2	p38α	p38β
AML-01	51	M	AML with inv(16)(p13.1q22); CBFB-MYH11	47,XY,inv(16)(p13q22),+22[23]/46,XY[2]	Yes	No	Yes	Yes
AML-02	73	M	AML with minimal differentiation	45,X,-Y[24]/46,XY[1]	Yes	No	No	No
AML-03	72	F	Therapy-related myeloid neoplasms	46,XX[25]	Yes	No	No	Yes
AML-04	76	F	AML with maturation	43,XX,-22,-18,der(12),-16[9]	No	No	No	Yes
AML-05	35	M	AML with inv(16)(p13.1q22); CBFB-MYH11	46,XY,inv(16)(p13q22)[17]	Yes	Yes	No	Yes
AML-06	60	F	Acute myeloid leukemia, NOS	47,XX,-3, del(5)(q13-q33),+8,+21,+21[6]/48,idem,+20[3]	Yes	No	No	Yes
AML-07	42	F	AML without maturation	46,XX[20]	Yes	Yes	No	Yes
AML-08	72	M	Acute monoblastic/monocytic leukaemia	46,XY[20]	Yes	Yes	No	Yes
AML-09	82	M	Acute myeloid leukemia, NOS	46,XY[25]	Yes	Yes	No	Yes
AML-10	33	M	Acute monoblastic/monocytic leukemia	46,XY[25]	Yes	Yes	No	Yes
AML-11	50	F	Acute myeloid leukemia, NOS	45,XX,-21,add(22)(p13)[26]/90<4n>,XXXX,t(11;17)(q13;p13),-21,-21,add(22)(p13)x2[24]	Yes	Yes	Yes	Yes
AML-12	53	M	Acute myeloid leukemia, NOS	46,XY,t(4;10)(p15;q21),add(19)(q13)[29]/46,XY[1]	Yes	No	No	Yes
AML-13	83	F	AML with myelodysplasia-related changes	45,XX,del(5)(q13q33),i(11)(q10),del(16)(q21),-17,add(17)(p13)[30]	No	Yes	No	Yes
AML-14	77	M	AML with minimal differentiation	45,X,-Y[24]46,XY[7]	Yes	Yes	No	Yes
AML-15	49	F	Therapy-related myeloid neoplasms	46,XX,t(2;12)(q31;p13),t(15;17)(q24;q21)[7]	Yes	Yes	No	Yes
AML-16	49	F	AML with inv(16)(p13.1q22); CBFB-MYH11	46,XX,inv(16)(p13q22)[9]	Yes	Yes	No	Yes
AML-17	25	F	Acute myeloid leukemia, NOS	ND	No	Yes	No	No
AML-18	62	F	AML with myelodysplasia-related changes	ND	No	No	No	No
AML-19	52	M	AML with maturation	86-94[7]/46,XX[13]	Yes	No	No	Yes
AML-20	42	M	AML with t(8;21)(q22;q22), RUNX1-RUNX1T1	45,X,-Y,t(8;21)(q22;q22)[1]/46,XY[99]	Yes	Yes	No	No
AML-21	77	M	AML with myelodysplasia-related changes	45,X,-Y[4]/44,X,-Y,add(7)(p22),-8[45]/46,XY[1]	No	No	No	Yes
AML-22	91	M	Acute myeloid leukemia, NOS	null	Yes	No	No	Yes
AML-23	88	M	Acute myeloid leukemia, NOS	ND	Yes	Yes	No	Yes
AML-24	62	F	Acute monoblastic/monocytic leukaemia	46,XX [20]	Yes	No	No	No
AML-25	88	F	Acute myeloid leukemia, NOS	ND	Yes	Yes	Yes	Yes
AML-26	60	F	AML with myelodysplasia-related changes	45,XX,-7[20]	Yes	Yes	No	No
AML-27	58	F	AML with myelodysplasia-related changes	46,XX/47,XX,+4[20]	Yes	Yes	No	Yes

Table S2. List of commercially available reagent used

Reagent	Ref#	Source	Concentration
NVP-BEZ 235	N-4288	LC Laboratories	1µM
SB203580	S3400	LC Laboratories	2.5µM
PH797804	S2726	Selleckchem Chemicals	250nM
SP600125	S7979	LC Laboratories	1µM
UO0126	U-6770	LC Laboratories	1µM
Cycloheximide	1810	Sigma	1µM
CX-4945	S2248	Selleckchem Chemicals	5µM
FTY720	S5002	Selleckchem Chemicals	5µM

Table S3. List of commercially available antibodies used

Target	Ref#	Source	Application
Alexa fluor 488 donkey anti-goat	A-11055	Invitrogen	IF
Alexa fluor 488 donkey anti-mouse	A21202	Invitrogen	IF
Alexa fluor 555 donkey anti-rabbit	A31572	Invitrogen	IF
Alexa fluor 568 donkey anti-mouse	A10037	Invitrogen	IF
Amersham ECL Mouse IgG, HRP-linked whole Ab (from sheep)	NA931	Amersham	WB
Anti-rabbit IgG, HRP-linked Antibody	7074	Cell signaling	WB
CK2 alpha	PA5-28686	Thermo	IF
CK2α	05-1431	MIllipore	WB
Donkey anti-goat IgG HRP affinity purified PAB	04HAF109	R&D systems	WB
GAPDH	9484	Abcam	WB
GRB2	610111	BD	WB
HSP27	2402	Cell signaling	WB
Lamin A/C	2032	Cell signaling	WB
p38α	9218	Cell signaling	IP, WB
p38α	sc-535	Santa Cruz Biotechnology	IF
p38β	2339	Cell signaling	IP, WB
p38β	AB183208	Abcam	IF
Phospho-CK2A (Thr360/Ser362)	SAB4300628	Sigma	WB
Phospho-HSP27 (Ser78)	E011247	Enogene	WB
PP2Ac	05-421	Millipore	IF, IP, WB
SET	sc-5655	Santa Cruz Biotechnology	IF, IP, WB
SETBP1	H00026040-B01P	Novus	IF
β-Actin	A5441	Sigma	WB
